# Identification of Diverse Toxin Complex Clusters and an eCIS Variant in Serratia proteamaculans Pathovars of the New Zealand Grass Grub (*Costelytra Giveni*) and Manuka Beetle (*Pyronota* Spp.) Larvae

**DOI:** 10.1128/Spectrum.01123-21

**Published:** 2021-10-20

**Authors:** Mark R. H. Hurst, Amy Beattie, Aurelie Laugraud, Richard Townsend, Lesley Sitter, Chikako van Koten, Lincoln Harper

**Affiliations:** a Resilient Agriculture, AgResearch, Lincoln Research Centre, Christchurch, New Zealand; b Bio-Protection Research Centre, Lincoln University, Christchurch, New Zealand; c Knowledge & Analytics, AgResearch, Lincoln Research Centre, Christchurch, New Zealand; d Curtin University, Centre for Crop and Disease Management, School of Molecular and Life, Bentley, Western Australia, Australia; University of Minnesota

**Keywords:** *Serratia proteamaculans*, *Serratia entomophila*, toxin complex, antifeeding prophage, tailocin, plasmid

## Abstract

The grass grub endemic to New Zealand, Costelytra giveni (Coleoptera: Scarabaeidae), and the manuka beetle, Pyronota festiva and P. setosa (Coleoptera: Scarabaeidae), are prevalent pest species. Through assessment of bacterial strains isolated from diseased cadavers of these insect species, 19 insect-active Serratia proteamaculans variants and a single Serratia entomophila strain were isolated. When independently bioassayed, these isolates differed in host range, the rate of disease progression, and 12-day mortality rates, which ranged from 60 to 100% of the challenged larvae. A *Pyronota* spp.-derived S. proteamaculans isolate caused a transient disease phenotype in challenged C. giveni larvae, whereby larvae appeared diseased before recovering to a healthy state. Genome sequence analysis revealed that all but two of the sequenced isolates contained a variant of the S. entomophila amber-disease-associated plasmid, pADAP. Each isolate also encoded one of seven distinct members of the toxin complex (Tc) family of insect-active toxins, five of which are newly described, or a member of the extracellular contractile injection (eCIS) machine family, with a new AfpX variant designated SpF. Targeted mutagenesis of each of the predicted Tc- or eCIS-encoding regions abolished or attenuated pathogenicity. Host-range testing showed that several of the S. proteamaculans Tc-encoding isolates affected both *Pyronota* and *C. giveni* species, with other isolates specific for either *Pyronota* spp. or *C. giveni.* The isolation of several distinct host-specific pathotypes of *Serratia* spp. may reflect pathogen-host speciation.

**IMPORTANCE** New pathotypes of the insect pathogen *Serratia*, each with differing virulence attributes and host specificity toward larvae of the New Zealand manuka beetle and grass grub, have been identified. All of the Serratia proteamaculans isolates contained one of seven different insect-active toxin clusters or one of three eCIS variants. The diversity of these *Serratia*-encoded virulence clusters, resulting in differences in larval disease progression and host specificity in endemic scarab larvae, suggests speciation of these pathogens with their insect hosts. The differing virulence properties of these *Serratia* species may affect their potential infectivity and distribution among the insect populations. Based on their differing geographic isolation and pathotypes, several of these *Serratia* isolates, including the manuka beetle-active isolates, are likely to be more effective biopesticides in specific environments or could be used in combination for greater effect.

## INTRODUCTION

The New Zealand grass grub, Costelytra giveni (Coleoptera: Scarabaeidae: Melolonthinae), formerly C. zealandica (1), is distributed throughout New Zealand, with the exception of the South Island’s west coast (Fig. S1) (2). The larvae feed on pasture roots, resulting in significant economic losses (3). Grass grub damage on dairy and meat farms has been estimated to cost NZ$215 to 585 million annually (3). Larvae of another beetle endemic to New Zealand, the manuka beetle (Pyronota festiva, P. laeta, and P. setosa; Coleoptera: Scarabaeidae: Melolonthinae), are smaller than their C. giveni counterparts but cause similar root damage in areas with light soils, particularly during pasture establishment (4, 5). *Pyronota festiva* and *P. laeta* are distributed throughout New Zealand, mainly in littoral zones such as sandy soils in coastal scrubland areas and adjacent to areas of native bush (6), while *P. setosa* is restricted to localized coastal pockets of sandy soil (6) (Fig. S1). The closely related endemic *Odontria* sp. (Coleoptera: Scarabaeidae: Melolonthinae) is distributed throughout the central and southern regions of New Zealand (7) and is not considered a pest species. *Costelytra giveni* and *Pyronota* spp. share a similar seasonal lifestyle, with larvae developing from late summer to mature by late winter, emerging as adults in late spring to early summer (4, 6). To date, amber disease caused by strains of Serratia entomophila and S. proteamaculans has been documented as the predominant disease of *C. giveni* larvae (2, 8).

Larvae challenged with amber-disease-causing *Serratia* strains cease feeding within 2 to 3 days, after which the dark larval gut is voided, resulting in the amber coloration (9). Amber disease takes 3 to 4 months to progress to the point where bacteria invade the hemocoel via the weakened gut lining, resulting in larval death (9). Relative to the approximate life span of the larvae (∼7 to 8 months), the disease period represents a significant part of the larval life stage and is therefore termed a chronic infection. Because of the chronic nature and low infective rate of the disease, S. entomophila is typically used as a preventative measure for grass grub management, with the *C. giveni*-specific S. entomophila-based product called Bioshield registered for the control of this insect pest (10).

The S. entomophila virulence determinants are encoded on an ∼153-kb plasmid called pADAP (amber-disease-associated plasmid) (11, 12). The first of these virulence determinants is the *sepABC* (S. entomophila pathogenicity) (13) insect-active toxin complex (Tc). Protein orthologs of *sepABC* were first described in the chromosome of the bacterium Photorhabdus luminescens (14) and have since been identified in chromosomes of Pseudomonas spp. (15), Xenorhabdus nematophilus (16), members of the genus *Yersinia* (17, 18), and Bacillus thuringiensis (19), among others. Typically, toxin complexes comprise three proteins, designated TcA, TcB, and TcC, which combine to form the final insect-active complex (20). The TcA component enables the delivery of the toxin effector and imparts host-range specificity (21, 22). The TcC component is a two-domain protein comprising a distinct C-terminal effector domain (13) and a conserved Rhs N-terminal domain, which, together with the TcB component, envelopes the TcC C-terminal effector (23), allowing its docking to the TcA delivery component. The *S. proteamaculans* strain 143 pADAP variant encodes Tc variants with divergent SepA and SepC orthologs SppA and SppC, respectively. Bioassays showed that only 60 to 70% of *C. giveni* larvae challenged with *S. proteamaculans* 143 succumbed to disease (12).

The second pADAP virulence determinant is a pyocin-like particle called the antifeeding prophage (Afp) (24, 25), variants of which have been identified in a diverse range of microbes (26, 27) and have recently been collectively termed extracellular contractile injection machines (eCIS) (28). Following ingestion by *C. giveni* larvae, the Afp is proposed to bind via its tail fibers to a yet-to-be-defined target cell, whereupon it contracts to deliver its protein effectors to the cell cytosol, resulting in cessation of feeding activity (24, 29). A second *Serratia* Afp variant, AfpX, encoded by *S. proteamaculans* subsp. *quinovora* strain AGR96X, has activity against both *C. giveni* and *Pyronota* spp. larvae. Differing from Afp, AfpX contains two tail-length termination proteins (AfpX16a and AfpX16b) and two predicted toxins (AfpX17 and AfpX18). Unlike S. entomophila, *S. proteamaculans* AGR96X invades the *C. giveni* larval hemocoel, where it rapidly multiplies to cause larval death within 5 to 12 days of ingestion (30). Of interest, both pathogenic plasmid-bearing and nonpathogenic isolates of S. entomophila and *S. proteamaculans* have been described (2, 8), with the bacteria typically found at levels of 1 × 10^3^ to 1 × 10^5^ bacteria per gram of New Zealand pasture soil (31).

Herein, we describe the isolation, identification, virulence gene characterization, and host-range testing of *Serratia* species isolated from field-collected *C. giveni* and *Pyronota* spp. larvae exhibiting a non-amber-disease phenotype and typified by a rapid progression of an amber/cream to brown and then to a blackened state. These potentially novel pathogens, specifically those species isolated from *Pyronota* spp., offer an opportunity to further our understanding of disease evolution and ecology and have the potential to be developed as biopesticides for specific environments.

## RESULTS AND DISCUSSION

### Defining species and genomic profiles of pathogens isolated from diseased *C. giveni* and *Pyronota* spp. larvae.

**FIG 1:**
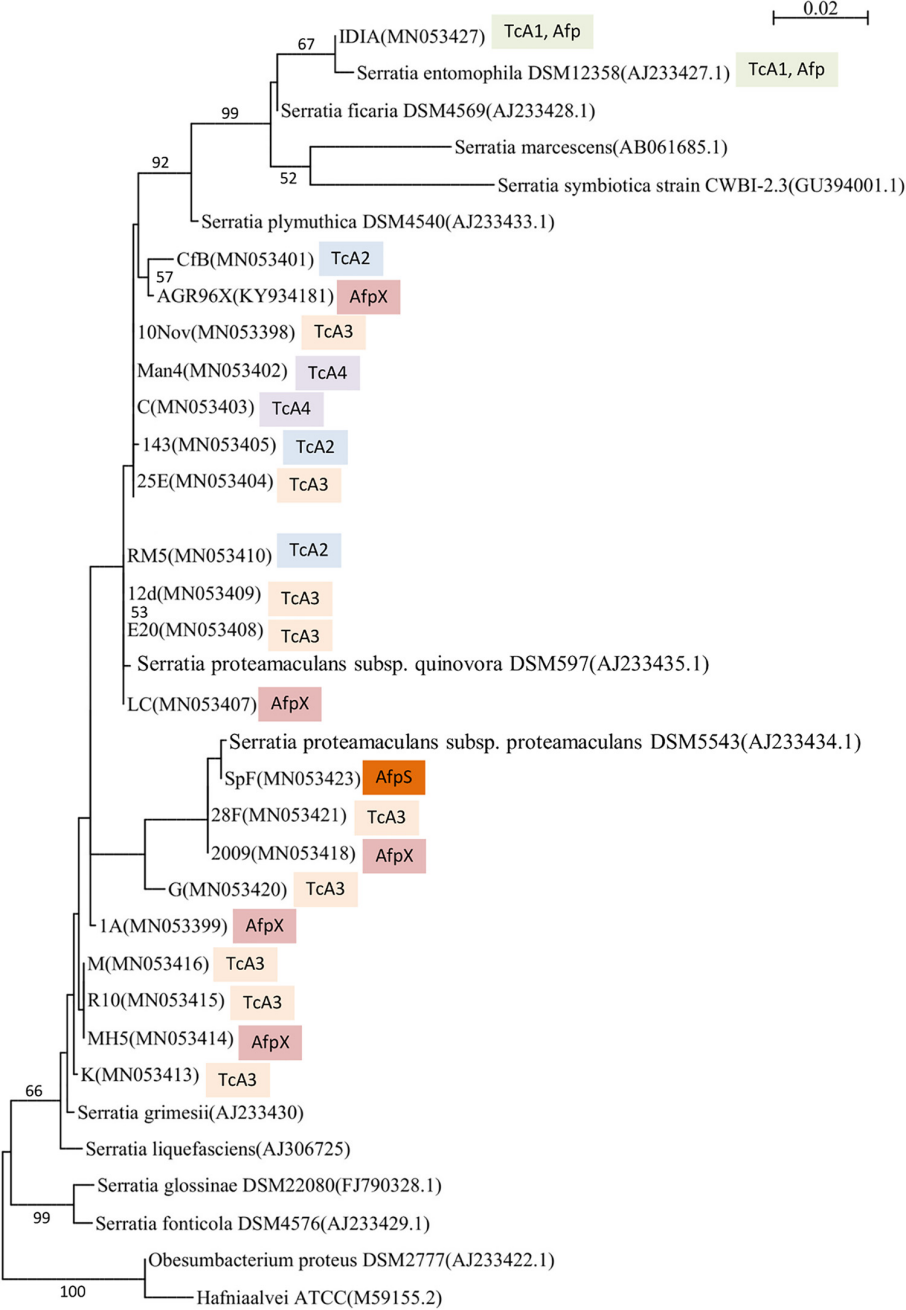
Maximum-likelihood phylogenetic tree based on the 16S rRNA gene sequences of *Serratia* isolates recovered in this study as well as the type species of closely related genera (16S rRNA GenBank accession numbers; color-coded TcA1-4 and eCIS cluster are indicated). The tree was generated using the GTR+I model (1,000 bootstraps). Numbers denote bootstrap values greater than 50%.

Grass grub and manuka beetle larvae exhibiting non-amber-disease states were sourced from several New Zealand locations (Table 1). From a total of 142 bacterial isolates recovered from these larvae and screened via bioassay, 19 isolates with distinct disease phenotypes and that differed in *tc* or eCIS (*afp*) PCR and amplicon sequence profiles (refer methods) were selected for further study (Table 1). Species identification was then carried out by 16S rRNA gene sequencing of each isolate. Phylogenetic analysis identified isolate IDIA as S. entomophila, with the other isolates identified as *S. proteamaculans*, *S. proteamaculans* subsp. *quinovora*, or *S. proteamaculans* subsp. *proteamaculans* (Fig. 1; Table 1).

**TABLE 1:** *Serratia* species, isolate name, larval and location source, predicted plasmid size, and GenBank accession number

Species	Isolate	Source	Source location	Predicted plasmid size (kb)^*a*^	Accession no.
S. entomophila	A1MO2	*C. giveni*	Mid Canterbury (low land)	153	AF135182
	IDIA	*C. giveni*	Lincoln (low land)	153	MT492124
*S. proteamaculans* subsp. *quinovora*	143	*C. giveni*	Methven, Canterbury (low land)	120, 90	FJ865409
	12D	*C. giveni*	Te Anau (hill country)	110	MN382395
	1A	*C. giveni*	Hororata (low land)	115	MN382405
	AGR96X	*C. giveni*	Lake Colleridge (hill country)	120	KU559315
	LC	*C. giveni*	Lake Colleridge (hill country)	120, 90	MN382404
	25E	*C. giveni*	Lake Colleridge (hill country)	110	MN382401
	20E	*C. giveni*	Lake Colleridge (hill country)	110	MN999470
	10NOV	*C. giveni*	Lincoln (low land)	110	MN382408
	CfB	*C. giveni*	Southland (hill country)	115	MN382399
	RM5	*Pyronota* spp.	Rangataikei (hill country)	90, 40	MN382391
	C	*Pyronota* spp.	Rangataikei (hill country)	120, 16	MN382398
	Man4	*Pyronota* spp.	Rangataikei (hill country)	120, 18, 16	MN382396
*S. proteamaculans* sp.	MH5	*C. giveni*	Lake Colleridge (hill country)	120	MW721595
	R10	*C. giveni*	Rangataikei (hill country)	110	MN382392
	M	*C. giveni*	Ohakune (hill country)	110	MN382394
	K	*C. giveni*	Ohakune (hill country)	110	MN382406
*S. proteamaculans* subsp. *proteamaculans*	2009	*C. giveni*	Lincoln (low land)	120	MN382403
	28F	*C. giveni*	Barlass Methven (hill country)	140	MN382400
	G	*C. giveni*	Lake Colleridge (hill country)	115	MN382407
	SpF	*C. giveni*	Hororata (low land)	120	MN382402

a Plasmid size estimates based on Kado and Lui (32) plasmid visualization; refer to Figure 2B. kb, kilobase.

To further differentiate the species, genomic BOX-repeat-based PCR (BOX-PCR) DNA fingerprinting analysis (32) of each of the isolates was performed, enabling isolates with differing genome compositions to be defined. Visual assessment of the resultant BOX-PCR profiles (Fig. 2A) revealed divergent DNA fingerprint profiles across the *S. proteamaculans* species isolates, indicative of differing genome composition, while the single S. entomophila isolate (IDIA) showed the same genomic BOX-PCR DNA fingerprint profile as S. entomophila A1MO2. This finding agrees with Dodd et al. (8), who identified high chromosomal divergence within *S. proteamaculans* but not S. entomophila. On the premise that previously documented *Serratia* virulence determinants (*sep*, *afp*, and *spp*) are plasmid encoded (8, 12), each isolate was independently screened for the presence of plasmids using the method of Kado and Liu (32). Through plasmid visualization, all isolates were found to contain at least one plasmid, with the plasmids of S. entomophila isolate IDIA and *S. proteamaculans* subsp. *proteamaculans* isolate 28F similar in size to pADAP (Fig. 2B; Table 1). To determine whether any of the observed plasmids were related to pADAP, we independently searched the genome sequences of each of the sequenced isolates for the pADAP *repA* gene. Using blastn similarity analysis, orthologs of *repA* were identified in 17 of the 19 isolates, with no *repA* orthologs identified in the *S. proteamaculans* subsp. *quinovora* isolates Man4 and C.

**FIG 2:**
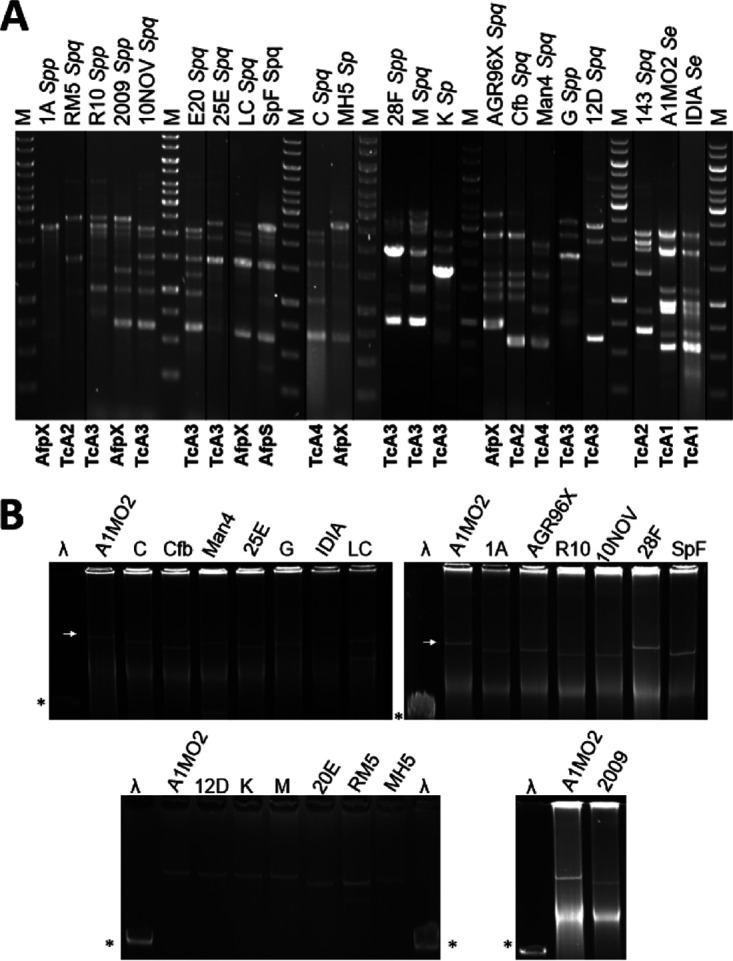
(A) Cropped BOX-PCR DNA fingerprint profiles of selected isolates. Symbols denote isolates with similar virulence clusters. Refer to Table 1 for isolate details. *Sp*, Serratia proteamaculans; *Spq*, *S. proteamaculans* subsp. *quinovora*; *Spp*, *S. proteamaculans* subsp. *proteamaculans*. M, GeneRuler 1-kb DNA ladder. Tc and eCIS cluster are indicated. (B) Kado Liu (32) plasmid visualization of selected isolates. White arrow denotes A1MO2 153-kb pADAP reference plasmid. λ, HindIII marker, with asterisk denoting 23-kb band.

### Host ranges of the isolates.

**FIG 3:**
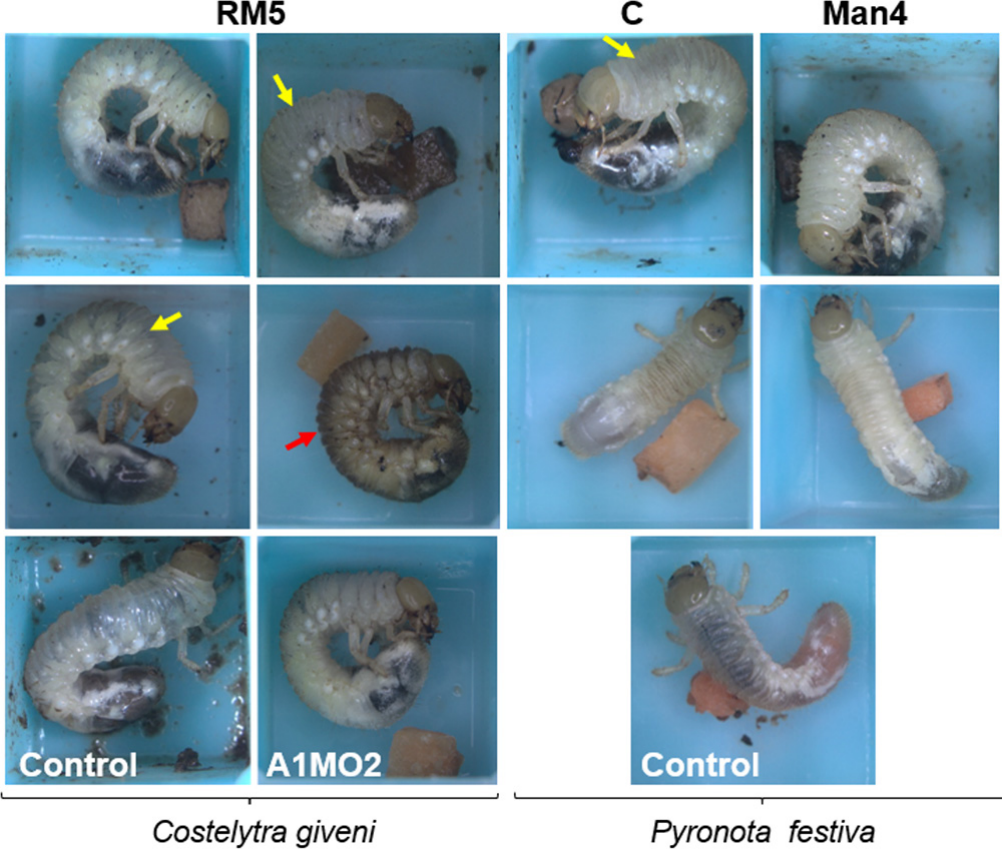
Photographs of *C. giveni* (RM5) and *P. festiva* (C, Man4) larvae 6 days postingestion of *S. proteamaculans* isolates RM5, Man4, or C. Man4 and C challenged *P. festiva* larvae exhibiting cream color relative to the untreated control. Yellow arrow denotes larval gut observed through larval cuticle integument. Red arrow, RM5 challenged *C. giveni* larvae transitioned from a cream to a brown color. The untreated *C. giveni* and its S. entomophila A1MO2 challenged counterpart are provided.

Challenge of *C. giveni* or *Pyronota* spp. larvae with each of the *Serratia* isolates revealed that disease progression times varied between isolates, with the exception of isolates C and Man4, where the challenged larvae remained cream in color (Fig. 3). Despite the differences in timing of disease progression, the susceptible challenged larvae underwent a similar disease-induced phenotypic progression, with larvae changing from a cream color to brown and then to a blackened state. Similar to *S. proteamaculans* strain 143 (12), the majority of the *S. proteamaculans* isolates did not cause 100% mortality in the challenged larvae (Table 2). Further, *S. proteamaculans* isolates 28F and MH5 exhibited variable virulence in *C. giveni* larvae in different bioassays, with the combined observed percent disease and mortality ranging from 58.3 to 100% for 28F and 50 to 100% for MH5 across several independent bioassays (Table S1). Of note, *C. giveni* larvae challenged with *Pyronota* spp.-derived isolate Man4 exhibited only partial disease symptoms, with some of the infected larvae transiently appearing opaque or amber but then recovering to a healthy state (Table 2; Fig. 3). This phenomenon has previously been noted in *C. giveni* larvae challenged with low concentrations of semipurified Sep proteins (33).

**TABLE 2:** Percent disease and mortality rates 12 days after maximum challenge of *C. giveni*, *P. sytosa*, and *P. festiva* larvae with the listed wild-type *Serratia* isolates

Isolate, differentiating virulence component (TcA; TcC; eCIS)^*a*^	*C. giveni*			*P. festiva*			*P. sytosa*		
	Disease (%) ± SE	Mortality (%) ± SE	Disease plus mortality (%) ± SE	Disease (%) ± SE	Mortality (%) ± SE	Disease plus mortality (%) ± SE	Disease (%) ± SE	Mortality (%) ± SE	Disease plus mortality (%) ± SE
S. entomophila									
A1MO2 (TcA1; TcC1)	57.1 ± 7.7	40.5 ± 7.7	97.6 ± 2.4	0	0	0	0	0	0
IDIA (TcA1; TcC1)	74.1 ± 6.0	22.2 ± 5.7	96.3 ± 2.6	0	0	0	0	0	0
*S. proteamaculans* subsp. *quinovora*									
143 (TcA2; TcC2a)	70.0 ± 15.3	0	70.0 ± 15.3	0	0	0	0	0	0
CFB (TcA2; TcC2a)	0	100	100	18.8 ± 10.1	75.0 ± 11.2	93.8 ± 6.3	13.3 ± 9.1	40.0 ± 13.1	53.3 ± 13.3
28F (TcA3; TcC2a)	14.3 ± 5.5	57.1 ± 7.7	71.4 ± 7.1	6.8 ± 3.8	2.3 ± 2.3	9.1 ± 4.4	0	0	0
20E (TcA3; TcC2a)	37.5 ± 8.7	31.3 ± 8.3	68.8 ± 8.3	0	0	0	0	0	0
25E (TcA3; TcC4)	52.2 ± 7.4	6.5 ± 3.7	58.7 ± 7.3	0	0	0	0	0	0
12D (TcA3; TcC4, TcC2a′)	72.2 ± 10.9	16.7 ± 9.0	88.9 ± 7.6	0	0	0	0	0	0
RM5 (TcA3; TcC4, TcC2a)	80.0 ± 13.3	20.0 ± 6.0	100	8.3 ± 8.3	25.0 ± 13.1	33.3 ± 14.2	0	37.5 ± 18.3	37.5 ± 18.3
10NOV (Tca3, TcC4)	4.5 ± 4.5	81.8 ± 8.4	86.4 ± 7.5	0	0	0	0	0	0
Man4 (TcA4; TcC2b, TcC3)	8.3 ± 5.8	12.5 ± 6.9	20.8 ± 8.5	25.0 ± 11.2	12.5 ± 8.5	37.5 ± 12.5	6.7 ± 6.7	13.3 ± 9.1	20.0 ± 10.7
C (TcA4; TcC2b, TcC3)	0	0	0	45.8 ± 10.4	29.2 ± 9.5	74 ± 9.0	0	8.3 ± 8.3	8.3 ± 8
AGR96X (AfpX)	10.7 ± 4.2	87.5 ± 4.5	98.2 ± 1.8	6.3 ± 4.3	90.6 ± 5.2	96.9 ± 3.1	15.0 ± 8.2	75.0 ± 9.9	90.0 ± 6.9
LC (AfpX)	10.7 ± 4.2	80.0 ± 13.3	90.7 ± 6.2	12.5 ± 5.6	87.5 ± 12.5	100	0	62.5 ± 18.3	62.5 ± 18.3
*S. proteamaculans* subsp. *proteamaculans*									
SpF (AfpS)	16.7 ± 11.2	75.0 ± 13.1	91.7 ± 8.3	0	100	100	0	50.0 ± 18.9	50.0 ± 18.9
2009 (AfpX)	4.5 ± 4.5	81.8 ± 8.4	86.4 ± 7.5	16.7 ± 11.2	75.0 ± 13.1	91.7 ± 8.3	0	100.0	100
1A (AfpX)	0	80.0 ± 13.3	80.0 ± 13	25.0 ± 13.1	75.0 ± 13.1	100	37.5 ± 18.3	37.5 ± 18.3	75.0 ± 16.4
G (TcA3; TcC4, TcC2a)	4.5 ± 4.5	68.2 ± 10.2	72.7 ± 9.7	0	0	0	0	0	0
*S. proteamaculans*									
MH5 (AfpX)	50.0 ± 10.4	41.7 ± 10.3	91.7 ± 5.8	50.0 ± 10.4	41.7 ± 10.3	91.7 ± 5.8	50.0 ± 10.4	41.7 ± 10.3	91.7 ± 5.8
R10 (TcA3; TcC2a)	30.0 ± 15.3	70.0 ± 15.3	100	20.0 ± 9.2	40.0 ± 11.2	60.0 ± 11.2	4.2 ± 4.2	0	4.2 ± 4.2
M (TcA3; TcC4)	11.8 ± 5.6	52.9 ± 8.7	64.7 ± 8.3	0	0	0	0	0	0
K (TcA3; TcC4)	50 ± 10.4	20.8 ± 8.5	70.8 ± 9.5	20.8 ± 8.5	79.2 ± 8.5	100	66.7 ± 9.8	33.3 ± 9.8	100

a The Tc (TcA and TcC) and the eCIS (Afp, AfpX, and AfpS) components are listed. Refer to Figure 4 for schematic of virulence clusters. SE, standard error.

Host-range testing of each of the isolates against selected scarab species showed that S. entomophila IDIA was virulent only in *C. giveni* larvae, while 13 *S. proteamaculans* isolates were active against both *C. giveni* and *Pyronota* spp. larvae (Table 2). Notably, *S. proteamaculans* isolate C, originally sourced from a *Pyronota* spp. larva, showed host specificity to *P. festiva* and *P. setosa*, with no activity toward *C. giveni* larvae. In contrast, *S. proteamaculans* isolate RM5, also sourced from a *Pyronota* spp. larva, was consistently active against *C. giveni* larvae, with only low activity against larvae of either *P. festiva* or *P. setosa*. None of the *Serratia* isolates were active against larvae of the closely related scarab *Odontria* sp. (data not shown). Further differences in host range of the isolates in relation to their associated predicted virulence clusters are discussed below.

### *In silico* analysis of putative virulence clusters.

To identify potentially novel virulence determinants, we sequenced the genomes of selected isolates. The resultant Illumina reads were then assessed for contigs with nucleotide similarity to the previously documented S. entomophila Sep/Afp- and *S. proteamaculans* Spp/AfpX-encoding clusters and new Tc/eCIS clusters when identified through the study. Through *in silico* analysis of the resultant assemblies, in addition to the previously documented Sep and Spp Tc clusters, five unique *tc* gene clusters (Fig. 4) and an additional eCIS AfpX variant, later named AfpS, were identified. Of the seven different Tc clusters identified across the *Serratia* isolates, four distinct *tcA* (*tcA1* to *tcA4*) and four distinct *tcC* (*tcC1* to *tcC4*) variants were identified (Fig. 4; Table 3). Further, three of the seven *tc* clusters encoded two different TcC proteins, with the remaining *tc* clusters encoding a single TcC protein (Fig. 4; Table 3).

**FIG 4:**
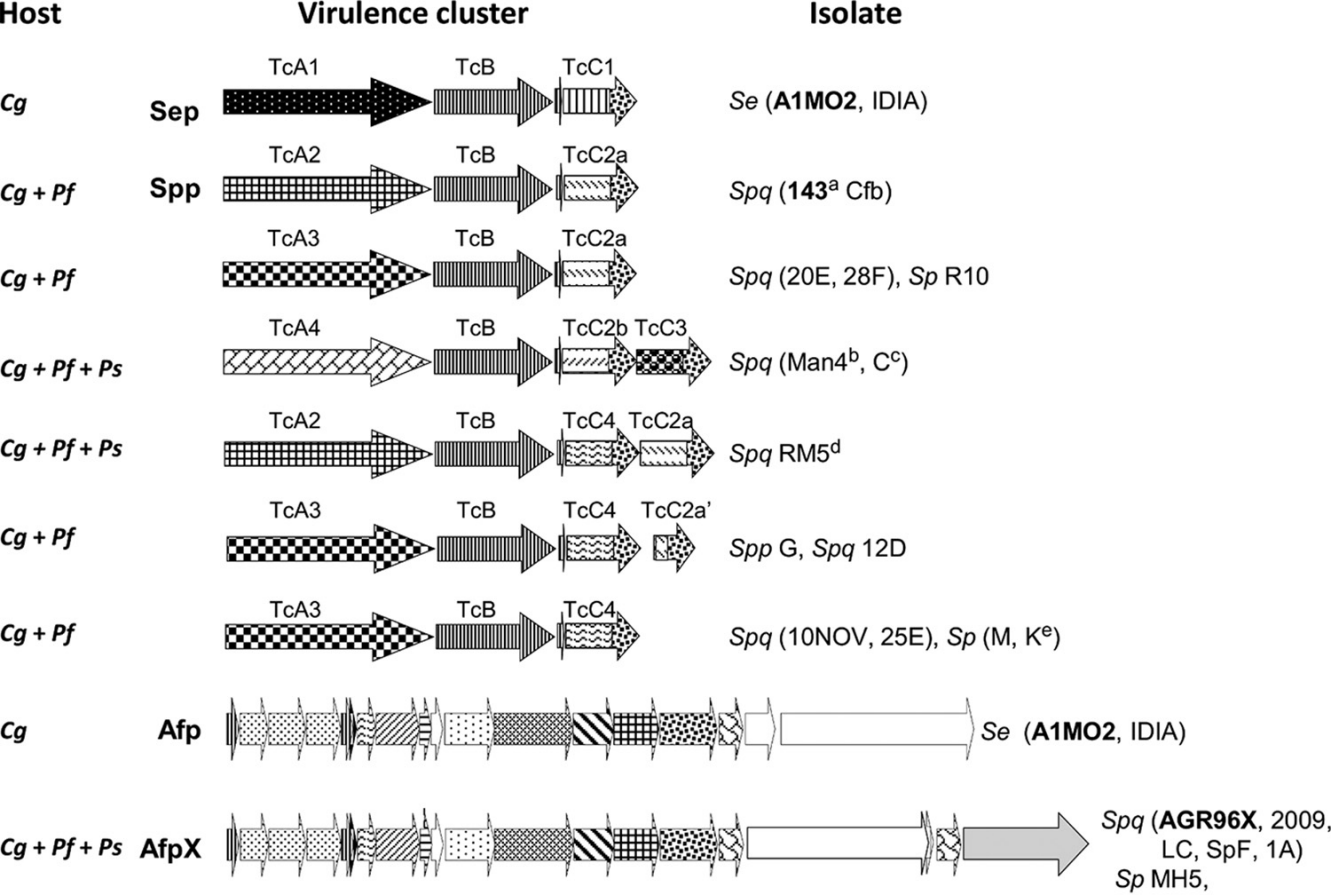
Schematic of the S. entomophila and *S. proteamaculans* Tc and Afp variants and their associated *C. giveni* (*Cg*), *P. festiva* (*Pf*), and *P. setosa* (*Ps*) host. Patinated arrows denote the Tc (TcA1 to 4, TcC1 to 4) variants and Afp variants identified in this study. *Sp*, Serratia proteamaculans; *Spq*, *S. proteamaculans* subsp. *quinovora*; *Spp*, *S. proteamaculans* subsp. *proteamaculans*. Isolate identifiers are listed. Isolate text bolding indicates the previously documented S. entomophila Sep and Afp and the *S. proteamaculans* isolate 143 Spp and AGR96X AfpX virulence-associated region. Isolate names are listed where superscript letters indicate (a) no activity against *Pyronota* species, (b) transient activity against *C. giveni*, (c) no activity against *C. giveni*, (d) transient activity against *Pyronota* species, (e) *Pyronota*-active. Refer to Table 3 for predicted TcA and TcC BLASTP similarities and Figure S2 for schematic of amino acid alignment of TcA1-TcA4 variants and their divergent regions.

**TABLE 3:** Amino acid similarities of products of predicted *Serratia* variable TcA and TcC orthologs to translated amino acid sequences in the database detected using BLASTP

ORF	No. amino acid residues	Isolates^*a*^	Locus tag	Species (amino acid length [bp])	Area of amino acid similarity^*b*^	Amino acid residues of target sequence	Ortholog accession no.
TcA							
TcA1, SepA	2,367	A1MO2, IDIA	SppA	*S. proteamaculans* (2,406)	(62/73)/2,443	1–2406	ACZ05627.1
TcA2, SppA	2,406	143, Cfb	SepA	S. entomophila (2,376)	(62/73)/2,443	6–2376	WP_010895734.1
			Toxin complex TcYF1	Yersinia frederiksenii (2,363)	(70/79)/2,426	6–2363	AAP48861.2
TcA3	2,499	R10, K, M, 28F, 20E, 12D, 25E, G, 10NOV	SppA	*S. proteamaculans* (2,406)	(90/92)/1,198	1–1198	ACZ05627.1
			Toxin complex	Xenorhabdus bovienii (2,506)	(44/59)/2,549	21–2506	WP_051861318.1
TcA4	2,409	Man4, C	SppA	*S. proteamaculans* (2,406)	(94/95)/2,406	1–2406	ACZ05627.1
TcC^*c*^							
TcC1, SepC	973	NA	Rhs repeat protein	Salmonella enterica (985)	(78/85)/985	1–985	EBR4566301.1
TcC2a, SppC	964	C1 (R10, 20E, Cfb, 143, 28F); C2 (12D, Rm5)	TcC component	Enterobacter sp. (956)	(66/77)/963	1–950	BBF83683.1
TcC2b	952	C1 (C, Man4)	SppC	*S. proteamaculans* (952)	(94/96)/952	1–952	ABB69941.1
TcC3	970	C2 (C, Man4)	Type IV secretion protein Rhs	Pantoea wallisii (977)	(66/77)/988	1–977	ORM74714.1
			SppC	*S. proteamaculans* (952)	(95/97)/680	1–680	ABB69941.1
TcC4	1,019	C1 (RM5, M, K, 12D, 25E)	Insecticidal toxin	Xenorhabdus ishibashii (1,020)	(54/67)/1,031	1–1020	PHM61563.1
			SppC	*S. proteamaculans* (952)	(98/98)/697	1–679	ABB69941.1

a Refer to Table 1 for isolate source.

b Area of amino acid similarity (% identity/% similarity over the indicated range of amino acid residues) in relation to sequence generated in this study. Similarities were considered significant if the BLASTP score exceeded e^−5^. Refer to Figure 4 for schematic of virulence clusters.

c C1 and C2 denote respective TcC operon components. NA, not available.

BLASTP analysis revealed that the *S. proteamaculans* TcA components showed the highest amino acid identity to the *S. proteamaculans* strain 143 SppA protein (TcA2) (Table 3). Within the TcA amino acid sequences, two regions of high amino acid, designated TcA regions (i) and (ii), were identified (Fig. S2). Relative to SppA, these regions span amino acid residues 306 to 429 and 1027 to 1658, respectively (Fig. S2). Interestingly, regions (i) and (ii) correspond to the structural regions of the *P. luminescens* strain PTC3 TcA homolog TcdA1, which is implicated in host specificity (22, 34), perhaps suggesting a similar role in the *Serratia* TcA1 to 4 variants. Phylogenetic analysis of the *Serratia* TcA components revealed that the *C. giveni-*specific S. entomophila SepA (TcA) protein was the most distantly related, with greater sequence divergence throughout the amino acid sequence compared with that of the other TcA proteins examined in this study (Fig. S2 and S3).

Amino acid alignments of the TcC1 to TcC4 variants, which differed in amino acid length (Table 3), revealed that TcC2a and TcC2b share 96% amino acid similarity. An 18-amino-acid variable region was identified in TcC2b and TcC1 spanning residues 145 to 163, upstream of the conserved glycine residue, preceding the divergent C-terminal effector (Fig. S4). TcC2a from isolate R10 showed a frameshift mutation at amino acid residue 186. The genomes of both isolates 12D and G were found to encode two TcC components: an N-terminal-truncated Tcc2a (Tcc2a′), in which the predicted C-terminal effector remains intact, and a Tcc4 component (Fig. 4; Table 3).

BLASTP analysis identified orthologs of some of the predicted TcC2 to 4 C-terminal effectors of unknown function in other entomopathogenic bacteria (Table 3). Phyre2 analysis of the S. entomophila TcC1 C-terminal region identified structural similarity to Pseudomonas aeruginosa type III secretion nucleotidyl cyclase toxin ExoY (Fig. S5), an effector that disrupts cell signaling, leading to cell death (35, 36).

Among the assessed Tc-encoding gene clusters, *tcB* and *gp55* (encoding a hypothetical protein) each shared >96% amino acid sequence identity with their respective S. entomophila orthologs (Fig. S6). The positioning of *gp55* between *tcB* and *tcC* is specific to *Serratia tc* clusters, with other *gp55* orthologs (Fig. S7) typically located adjacent to open reading frames (ORFs) encoding phage-like proteins (Table S2).

### Validating the role of *Serratia* toxin complexes in virulence.

To validate the role of the identified Tc clusters, the *tc* associated regions from each of the various *S. proteamaculans* and S. entomophila isolates were independently mutated to delete the 3′ region of *tcA* and the 5′ region of *tcB* (see Materials and Methods). The resultant mutants (Table S5) were then assessed by bioassay. With the exception of *S. proteamaculans* isolate 20E and S. entomophila isolate IDIA (encoding Sep and Afp homologs), *C. giveni* larvae independently challenged with mutated strains remained healthy, validating the role of these Tc components in the virulence of the wild-type isolates (Table 4).

**TABLE 4:** Percent disease and mortality rates at 12 days postchallenge of *C. giveni* larvae with the listed isolates at approximately 4 × 10^8^ cells per carrot

Treatment^*a*^	Diseased (%) ± SE	Mortality (%) ± SE	Disease + mortality (%) ± SE
Control	0	0	0
28F	83.3 ± 7.8	12.5 ± 6.9	95.8 ± 4.2
28FΔTCAB	0	0	0
20E	37.5 ± 10.1	33.3 ± 9.8	70.8 ± 9.5
20EΔTCAB	20.8 ± 8.5	16.7 ± 7.8	37.5 ± 10.1
RM5	80.0 ± 13.3	20.0 ± 6.0	100
RM5ΔTCAB	8.3 ± 5.8	0	8.3 ± 5.8
CfB	62.5 ± 10.1	37.5 ± 10.1	100
CfbΔTCAB	0	4.2 ± 4.2	4.2 ± 4.2
Man4	16.7 ± 7.8	4.2 ± 4.2	20.8 ± 8.5
Man4ΔTCAB	4.2 ± 4.2	8.3 ± 5.8	12.5 ± 6.9
R10	30 ± 15.3	50 ± 16.7	80 ± 13.3
R10ΔTCAB	0	0	0
C	16.7 ± 7.8	4.2 ± 4.2	20.8 ± 8.5
CΔTCAB	0	4.2 ± 4.2	4.2 ± 4.2
M	25.0 ± 9.0	58.3 ± 10.3	83.3 ± 7.8
MΔTCAB	4.2 ± 4.2	0	4.2 ± 4.2
25E	11.8 ± 5.6	52.9 ± 8.7	64.7 ± 8.3
25ΔTCAB	0	0	0
G	4.5 ± 4.5	68.2 ± 10.2	72.7 ± 9.7
GΔTCAB	0	0	0
10NOV	4.5 ± 4.5	81.8 ± 8.4	86.4 ± 7.5
10NOVΔTCAB	0	4.2 ± 4.2	4.2 ± 4
12D	30.0 ± 15.3	50.0 ± 16.7	80.0 ± 13.3
12DΔTCAB	0	0	0
K	50.0 ± 10.4	20.8 ± 8.5	70.8 ± 9.5
KΔTCAB	4.2 ± 4.2	0	4.2 ± 4.2
IDIA variants			
Control	0	4.2 ± 4.2	4.2 ± 4.2
IDIA	54.2 ± 10.4	45.8 ± 10.4	100
IDIAΔAFP	58.3 ± 10.3	41.7 ± 10.3	100
IDIAΔTC	62.5 ± 10.1	37.5 ± 10.1	100
IDIAΔAfpΔTC	37.5 ± 10.1	8.3 ± 5.8	45.8 ± 10.4
AfpX variants			
1A	4.2 ± 2.9	89.6 ± 4.4	93.8 ± 3.5
1AΔ1516a	5.6 ± 3.8	8.3 ± 4.6	13.9 ± 5.8
2009	4.2 ± 2.9	83.3 ± 5.4	87.5 ± 4.8
2009Δ1516a	5.6 ± 3.8	2.8 ± 2.7	8.3 ± 4.6
LC	2.1 ± 2.1	89.6 ± 4.4	91.7 ± 4
LCΔ1516a	5.6 ± 3.8	11.1 ± 5.2	16.7 ± 6.2
MH5	0	80.0 ± 13.3	80.0 ± 13.3
MH5Δ1516a	0	0	0
SpF	91.7 ± 8.3	8.3 ± 8.3	100
SpFΔ1516a	8.3 ± 4.6	2.8 ± 2.7	11.1 ± 5.2
AGR96X	13.9 ± 5.8	80.6 ± 6.7	94.4 ± 3.9
AGR96X Δ1516a	4.2 ± 4.2	0	4.2 ± 4.2
Control	0	1.7 ± 1.7	1.7 ± 1.7

a Δ denotes internal deletion, refer to Table S5 for strain details.

Although they encode the same virulence determinants, maximum challenge bioassays of S. entomophila isolates A1MO2 and IDIA revealed that the onset of disease symptoms in IDIA-challenged larvae was more rapid than that observed in A1MO2-challenged larvae (Table S4). In addition, bioassays of the IDIAΔTCAB strain revealed that the challenged *C. giveni* larvae took on an amber coloration (Table 4), while larvae challenged with A1MO2ΔTCAB remained healthy but ceased feeding. Further, bioassays of the IDIAΔTCΔAFP mutant showed that while its virulence was attenuated, a basal level of activity against grass grub larvae remained (Table 4). Based on this information, it is plausible that isolates IDIA and 20E encode an additional, yet-to-be-determined virulence factor.

The loss of activity of the TcAB (ΔTCAB) derivatives supports a role for TcA proteins, and potentially their TcC effectors, in host specificity. With reference to Figure 4 and Table 2, the S. entomophila isolates encoding TcA1 (SepA) were *C. giveni* specific. Though causing low activity, isolates encoding the TcA4 variant appeared specific to *P. festiva* and *P. setosa* with only transient (Man4) or no (isolate C) activity against *C. giveni* larvae (Table 2). With the exception of isolate 143, isolates encoding TcA2 or TcA3 were active against *C. giveni* and *P. festiva* but not *P. setosa*, with isolate RM5 (TcA2) demonstrating low activity against both *P. festiva*- and *P. setosa*-challenged larvae. The *S. proteamaculans* isolates, Cfb and 143, encoded the same virulence cluster but exhibited divergent host range. Serratia proteamaculans isolate K, although encoding the same Tc cluster as the non-*Pyronota*-active isolates 10NOV, M, and 25E, was active against both *P. festiva* and *P. setosa*. These results allude to the potential regulation of virulence or additional genome-encoded factors required in the manifestation of disease.

**FIG 5:**
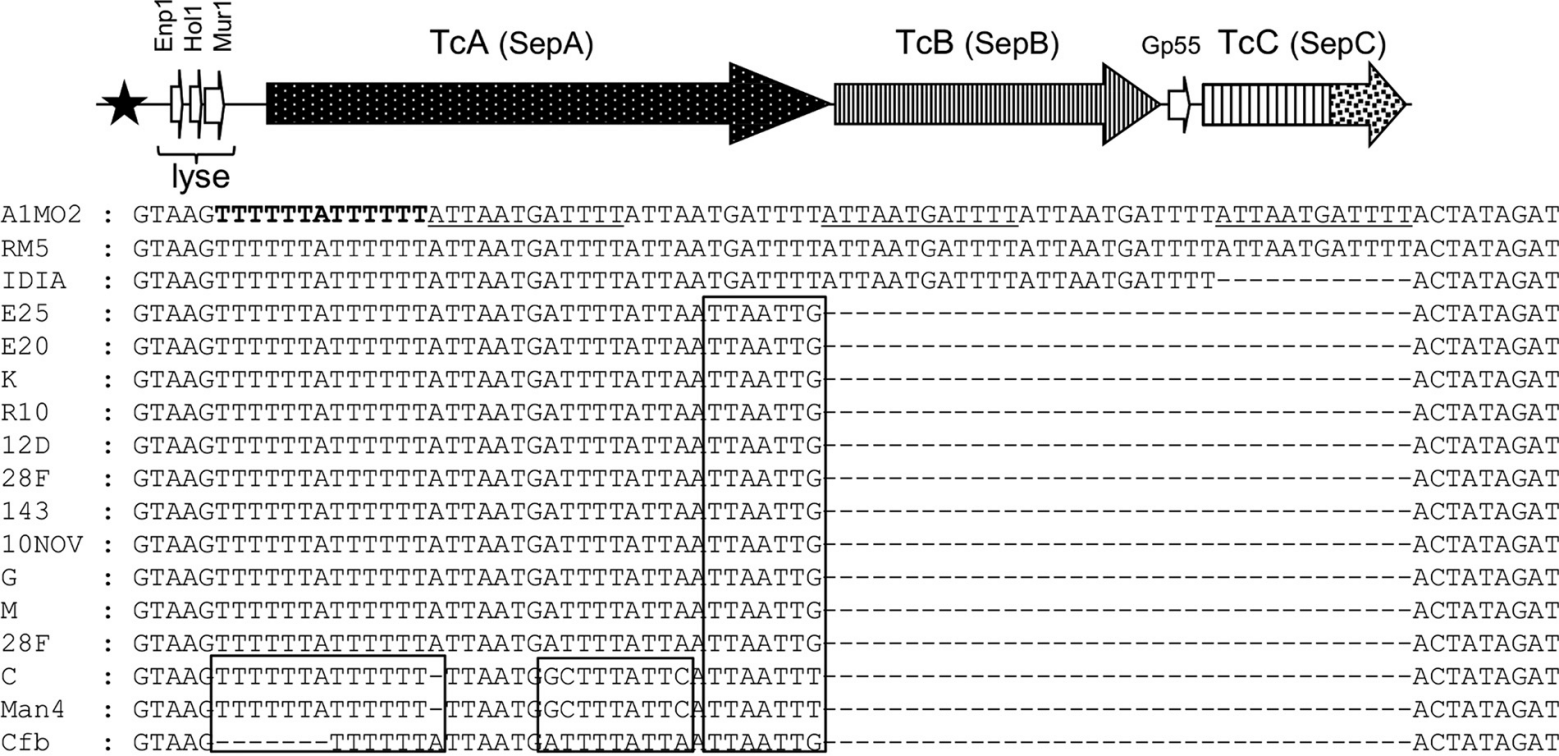
Schematic of the *Serratia* Tc-encoding cluster and its 12-bp element-associated region. Predicted lysis cassette “lyse” located 5′ of the *tc* cluster is indicated. Filled star denotes the location of the 12-bp element-associated region. Nucleotide sequence alignment of the divergent 12-bp element-associated region with reference to S. entomophila A1MO2 element comprising a dyad symmetry (bold) and 12-bp element (underscore). Boxed outline denotes nucleotide differences in the repeat-associated region.

Guided by this information, we assessed nucleotide regions 5′ of a predicted cell lysis cassette (14), along with its associated Tc-encoding region, in each of the Tc-encoding isolates. Differences were observed among the isolates in the DNA repeat region, previously documented in S. entomophila A1MO2 (13), which comprises five 12-bp repeat elements 3′ of an 11-bp region of dyad symmetry (Fig. 5). Serratia entomophila isolate IDIA contained four repeat elements, while the remaining Tc-encoding *S. proteamaculans* isolates contained a single 12-bp element. Nucleotide alterations in the 11-bp dyad region were also identified in the *S. proteamaculans* isolates Cfb, C, and Man4 (Fig. 5).

### Validating the role of *Serratia* eCIS Afp (tailocin) in virulence.

*In silico* analysis of the non-Tc-encoding isolates (2009, LC, MH5, 1A, and SpF) revealed the presence of eCIS Afp orthologs sharing >98% nucleotide sequence identity with the AGR96X AfpX-encoding cluster (30). The exception was AfpS from *S. proteamaculans* subsp. *proteamaculans* isolate SpF, wherein AfpS10, AfpS11, AfpS12, and AfpS13 showed only 98, 88, 89, and 72% amino acid sequence similarity to their respective AfpX10, AfpX11, AfpX12, and AfpX13 proteins. The S. entomophila Afp orthologs comprise the proteins to which the Afp13 tail fiber attaches (25). Amino acid sequence alignments of the Afp13, AfpX13, and AfpS13 tail fiber proteins identified differences in the number of conserved tail fiber shaft repeats (Fig. S8A and B). While slightly divergent from the *C. giveni*-specific Afp, the predicted AfpX and AfpS13 receptor-targeting knob regions were similar (Fig. S8C).

Despite their differing genomic BOX-PCR DNA fingerprinting profiles (Fig. 1A) and bioassay efficacies (Table 2), each of the AfpX-encoding variants (AGR96X, 2009, LC, MH5, and SpF) harbored an ∼120-kb plasmid (Table 1; Fig. 1B). Except for MH5 (Table S1), these isolates were the most virulent among those tested, causing ≥90% mortality in the challenged larvae within 5 to 12 days of ingestion (Table 2). Similar to AGR96X, host-range testing of these isolates showed them to be active against *C. giveni*, *P. festiva*, and *P. setosa* larvae but not against larvae from the closely related scarab genus *Odontria* (data not shown). Isolate SpF was highly active against *P. festiva* larvae, demonstrating 100% mortality at 12 days postchallenge (Table 2). The nucleotide regions 5′ of *afp*, *afpX*, and *afpS* and the regions 5′ of the *amb*2 locus (implicated in Afp gene regulation [29]) in the respective isolates were identical (data not shown), suggesting that these regions are not involved in the altered virulence phenology of the isolates. Targeted mutagenesis of the *afpX15* to *afpX16a* component of the *afpX*-like gene clusters significantly attenuated activity against challenged *C. giveni* larvae, where, with the exception of MH5, a low level of disease in challenged larvae was observed (Table 4).

### Pathotype and biogeography.

**FIG 6:**
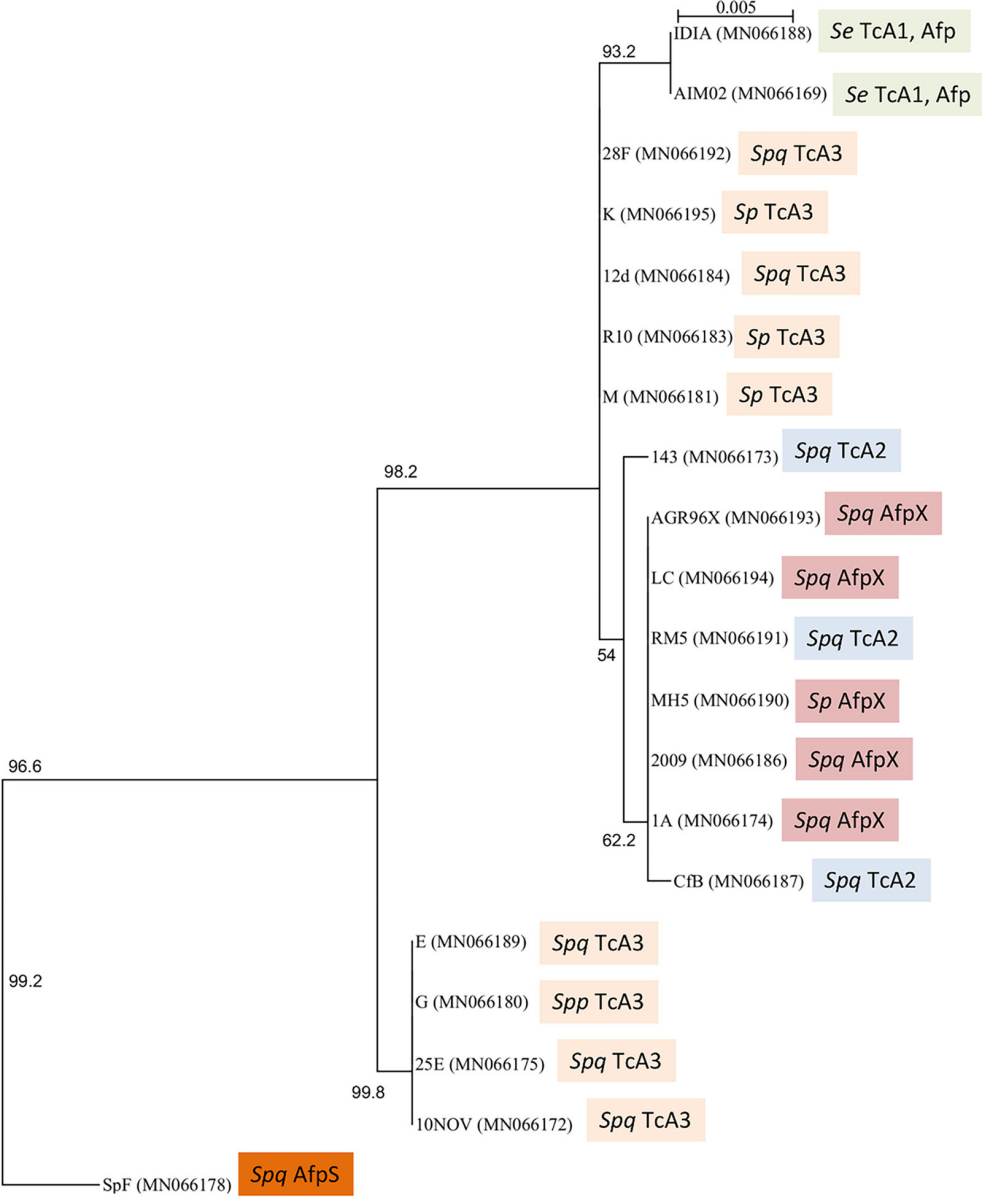
Maximum-likelihood phylogenetic tree based on the S. entomophila and *S. proteamaculans repA* gene sequences, generated using the GTR+I model (1,000 bootstraps). Numbers denote bootstrap values greater than 50%. *Sp*, Serratia proteamaculans; *Spq*, *S. proteamaculans* subsp. *quinovora*; *Spp*, *S. proteamaculans* subsp. *proteamaculans*. *repA* GenBank accession numbers; color-coded TcA1-4 and eCIS cluster are indicated.

To define the possible origin of the *Serratia* virulence determinants, we compared the nucleotide sequences of the 16S rRNA gene, the pADAP replication *repA* gene, and components of the *tc* (excluding *S. proteamaculans* isolates C and Man4 encoding a non-pADAP associated TcA4 variant) and eCIS *afp*-like gene clusters from all of the bacterial isolates. Based on 16S rRNA and *repA* sequence analysis, the TcA2-encoding isolates RM5 and Cfb and eCIS *afpX* clusters (excluding the isolate SpF, *afpS* eCIS variant) resided on *S. proteamaculans* or *S. proteamaculans* subsp. *quinovora* plasmids sharing high *repA* nucleotide sequence identity. TcA3 falls into two *repA* subgroups shared across the three *S. proteamaculans* species, and the more distinct TcA1 is placed within the *C. giveni*-specific S. entomophila (Fig. 6).

Unlike the Afp- and Sep-encoding S. entomophila isolates, none of the *S. proteamaculans* isolates assessed in this study encoded both the *tc* and *afp* gene clusters, suggesting that the more rapid onset of disease induced by these isolates may override the requirement for a second virulence determinant. It remains undetermined as to why one type of virulence cluster or *Serratia* species has not become dominant in the *C. giveni* and *Pyronota* system, nor is it clear why there are only two main Afp variants (AfpS shows amino acid sequence divergence in the tail fiber region; Fig. S8) but several Tc orthologs and associated TcC effectors, with some *tc* clusters encoding two TcC effectors. Multiple Tc clusters are found in the genomes of Pseudomonas species (15), *P. luminescens* (37), and *Yersinia* species (17, 29), among others (38). Of these species, the *Serratia* Tc-encoding regions are most closely related to Tc clusters from *Yersinia* and *Rhanella* (38). For example, the non-*C. giveni*-active Yersinia frederiksenii isolate 49 plasmid py49 contains *tcYF1* and *tcYF2* (8), which share >80% amino acid identity with their respective SepA and SepB orthologs from *Serratia* (Fig. S3 and S6). The diversity of Tc-encoding clusters across *Proteobacteria* signifies that the Tc proteins play a key role in the ecology of these bacteria (38).

The wide range of different *tc* clusters in part parallels the diversity of three-domain Cry toxins, where the various composite domains are proposed to have evolved at their own rates (39), with greater variability in domains implicated in host-targeting and effector type (40). Similarly, the *tc* clusters identified in the current study differed in their predicted TcA host-targeting [regions (i) and (ii)] and TcC effector components. The diversity of *tc* clusters may reflect an enhanced evolutionary capacity for these regions to acquire or exchange additional *tcC* components, as originally proposed by Hill et al. (41). This selective process in turn may be driven by a pathogen-host arms race as has been proposed in other effector systems (42). Based on studies of other bacterial effectors (43–45), the different *Serratia-*encoded TcC effectors, when delivered, will likely have differing subcellular effects, leading to either subtle or acute phenotypes that may be in tune with the pathogen’s lifestyle.

Through the course of this study, differences in both host range and virulence capacity of isolates encoding the same virulence determinants (Cfb and 143) were observed, while isolates such as 28F and MH5 exhibited variable pathogenicity over different bioassays. This, together with the identification of different DNA repeat elements 5′ of Tc-encoding gene clusters and the altered phenotypes of the *afp* and *sep* mutant S. entomophila isolates A1MO2 and IDIA (Table S4), alludes to chromosomally encoded virulence determinants and/or virulence regulation as key components of these processes.

In relation to the biogeography and insect host range of the isolates identified in this study, the S. entomophila isolates encoding the Afp and Sep amber-disease-associated virulence determinants along with other *Serratia* Tc-encoding variants were typically isolated from low-lying regions of modified pasture inhabited by *C. giveni* and/or *Pyronota* species (Table 1; Table S3) (2, 8). The most virulent Tc-encoding *S. proteamaculans* isolates (28F and Cfb), which were active against *C. giveni* and *Pyronota* spp., were isolated from hill country areas (Table 1). In contrast, the most virulent isolates encoding AfpX-like variants (MH5, LC, 1A, 2009, SpF) were recovered from modified pastures in both lowland and hill country areas of Canterbury, New Zealand (Table 1). Of note, *S. proteamaculans* isolate MH5 was isolated at the same time and from the same field site (Lake Coleridge region, New Zealand; Table 1) as *S. proteamaculans* isolate AGR96X and the broad host-range entomopathogen Y. entomophaga (46), indicative of a multientomopathogen complex at this location. No Tc-encoding *Serratia* isolates were identified at this site. The high number of *S. proteamaculans* isolates with activity against both *C. giveni* and *P. festiva* likely reflects the codistribution of these insect species (Fig. S1). Historically, *C. giveni-*specific S. entomophila and *S. proteamaculans* have been isolated throughout New Zealand from *C. giveni* larvae exhibiting chronic amber disease symptoms (Table S3) (2, 8), an isolation regime preferentially selecting for *C. giveni* pathogens such as S. entomophila.

In relation to disease epidemiology, the process of pathogen infectivity will be influenced by host larval population density. The persistence of chronic pathogens such as S. entomophila is aided by larval movement through its environment, affording the pathogen greater opportunity to infect other larvae. The prevalence of amber-disease-causing *Serratia* increases along with an increasing *C. giveni* larval population for 3 to 5 years, after which the buildup of amber disease causes the larval population to collapse and a resulting decline in amber-disease-causing bacteria (9). In the absence of a susceptible host population, levels of disease-causing strains fall, with the majority of *Serratia* isolates being nonpathogenic (31).

The epidemiological aspects of non-amber-disease *S. proteamaculans* pathotypes remain undefined. It is likely that faster acting pathogens such as *S. proteamaculans* AGR96X may be predisposed to localized outbreaks characterized by a “boom to bust” life cycle (47) or, alternately, suited to cooler climes where reduced larval metabolism limits larval movement. This may mean that unlike amber-disease-causing isolates, rapid-killing pathogens have less capacity to spread and persist in subsequent years (47, 48). In addition to these variables, though it may reflect differences in larval physiology or genetics, the capacity of some of the *Serratia* isolates to cause disease in only 60 to 90% of the challenged larvae may ensure the long-term persistence of the pathogen by maintaining host availability in subsequent years.

Interestingly, despite the relatively wide geographic spread of the sampling locations in this study, only S. entomophila and *S. proteamaculans* isolates were recovered from the diseased *C. giveni* and *Pyronota* spp. larvae. These scarab species evolved after New Zealand split from Australia some 82 million years ago (49). To date, scarab-active *Serratia* isolates have been documented only in New Zealand, with no scarab-active bacterial pathogens yet described in Australia. This, combined with the host specificity and the unique virulence determinants of the S. entomophila and *S. proteamaculans* isolates, suggests that this host-pathogen relationship may have coevolved.

The isolation of different *Serratia* strains encoding different Tc and eCIS clusters may reflect their requirement in a particular environment. Accordingly, these isolates offer the potential for the development of biopesticides that are more active in specific environments. Further, combining isolates with different virulence properties, such as S. entomophila and the more rapid-killing *S. proteamaculans* isolates identified in this study, may afford greater control of the insect host (47). The isolation of new *Pyronota*-active Tc-encoding *Serratia* species offers additional measures for the control of this species. To understand the differing virulence properties of several of the bacterial isolates identified here, additional studies on the insect host, pathogen distribution, genetics, and virulence gene regulation are required.

## MATERIALS AND METHODS

### Bacterial strains, culture methods, and pathogen isolation.

Wild-type bacterial isolates and defined virulence characteristics are listed in Table 1. Modified bacterial strains and plasmids used in this study are listed in Table S5. Bacteria were cultured in Luria-Bertani (LB) broth or on LB agar plates at 30°C (S. entomophila and *S. proteamaculans*) or 37°C (Escherichia coli). Cultures were incubated with shaking at 250 rpm in a Ratek orbital incubator. Antibiotics were included when required at the following concentrations (μg/ml). *Serratia* spp.: kanamycin (100), tetracycline (30); E. coli: kanamycin (50), tetracycline (15), ampicillin (100). The culture medium of E. coli strain ST18 was supplemented with 50 μg/ml 5-aminolevulinic acid. Potential bacterial pathogens were isolated from diseased or dead larvae as described previously (33).

### DNA isolation and manipulation.

Standard DNA techniques were performed as described by Sambrook et al. (50). PCR primers used in this study, along with the sizes of the corresponding amplicons, are listed in Table S6. PCR primer sets were designed manually or using Primer3 (51). DNA used as the template for BOX-PCR and for genome sequencing was independently prepared from each isolate using a Bioline (London, United Kingdom) Isolate II genomic DNA kit (BIO-52066) as per the manufacturer’s instructions. Plasmid DNA and PCR products were independently purified using High Pure plasmid isolation and High Pure PCR product kits (Roche Diagnostics GmbH, Mannheim, Germany), respectively. Plasmids were electroporated into E. coli and *S. proteamaculans* strains using a Bio-Rad gene pulser (25 μF, 2.5 kV, and 200 Ω) (52). Visualization of large plasmid DNA was performed using the method of Kado and Liu (32). All plasmids constructed in the study were validated by DNA sequencing using appropriate vector-specific primers (Table S6).

### Rationale for identifying new strains for genome sequencing.

Initially, primer sets (Table S6) were designed to amplify regions of the pADAP *rep*A gene (12) and conserved regions of the known S. entomophila and *S. proteamaculans* virulence (Tc, *sep* [13], *spp* [12] and the eCIS *afp* [24], *afpX* [30]) gene clusters. The resultant amplicons from PCR-positive isolates were then sequenced and assembled against the relevant target gene sequences, where if the DNA was not 100% identical to that of the virulence-associated amplicon and the translated amino acid sequence was orthologous to a Tc or Afp component, the genome of that isolate was sequenced. As new genomes were sequenced, additional virulence gene-specific primer sets were designed, enabling the detection of similar virulence clusters in other isolates and the removal of those isolates from genome sequencing.

### DNA sequencing and *in silico* analysis.

Whole-genome sequencing of individual bacterial isolates was carried out using the Illumina HiSeq 2500 system by Macrogen Sequencing Services (Macrogen Inc., Seoul, Republic of Korea). For each isolate, DNA sequences were trimmed using Trim_Galore (http://www.bioinformatics.babraham.ac.uk/projects/trim_galore/) and assembled using A5-miseq (53). When required, sequencing gaps were closed using Sanger sequencing of PCR amplicons and the resultant sequences were assembled using Geneious version 8.1.5 (54). Open reading frames (ORFs) were defined using Geneious version 8.1.5.

Databases at the National Center for Biotechnology Information were searched using BLASTN, BLASTX, BLASTP (55), and Phyre2 (56). Amino acid and 16S rRNA gene sequences were aligned using Muscle (57). PhyML (58) was used to produce phylogenetic trees using a maximum-likelihood approach with 1,000 bootstrap resamples. Amino acid preferences were determined using WebLogo 3 (59). The nucleotide sequences of the virulence-associated regions, the 16S rRNA gene, and the pADAP *repA* gene from each of the *Serratia* isolates identified in this study have been deposited in GenBank under the accession numbers listed in Table 1 and Figures 1 and 6.

### BOX-PCR.

BOX-PCR fingerprint profiling was undertaken using purified genomic DNA and a single A1R primer as outlined previously (60), using ReddyMix PCR master mix (Thermo Scientific, Waltham, United States) according to the manufacturer’s instructions. Thermal cycler parameters were as follows: 94°C for 15 min, followed by 30 cycles of 94°C for 15 s, 53°C for 30 s, and 68°C for 8 min, and a final extension step of 68°C for 10 min. DNA fragments were then separated on a 1% agarose gel.

### Targeted mutagenesis of the *tcAB*, *afp15* to *16* regions.

Based on nucleotide sequence alignments of the Tc-encoding regions from the various *S. proteamaculans* and S. entomophila isolates, a 4,044-bp fragment sharing greater than 97% nucleotide identity across each of the sequenced Tc clusters, deleting 293 bp of the *tc*A (*spp*A) end to 19 bp 3′ of the *tc*B (*spp*B) initiation codon, was PCR amplified, and the resultant amplicon, TCAB (Table S6), was cloned into pGEM-T easy (Promega corporation) to form pGEM143A. pGEM143A was then digested with MscI and SmaI, releasing a 293-bp fragment into which the EcoRV-flanked amplicon SPRV (Table S6) was ligated to form pGEM143SPRV. The vector pGEM143SPRV was then digested with BamHI and the released fragment was ligated into the analogous site of pJP5603 (61) to form pJP143TCAΔBSP.

Based on the shared nucleotide identity, for targeted mutation of the AfpX- and AfpS-encoding strains, the previously constructed vector pJP5608ΔAFP1516a (Table S5) was used to inactivate *afp15*, encoding the predicted AfpX15 ATPase chaperone and the N terminus region of AfpX16a, both of which are required for Afp assembly (30). For targeted mutation of the S. entomophila *afp* homologous gene cluster in IDIA, the previously constructed plasmid pMH52ΔBglII (Table S5) was used to generate an S. entomophila Afp deletion variant missing a 16,064-kb BglII fragment encompassing the Afp genes *afp*2 to *afp*15, as outlined previously (24).

Antibiotic marker genes were recombined into pADAP variants via conjugation of E. coli ST18 containing either pJP5608ΔAFP1516a or pJP143TCAΔBSP with the appropriate recipient *S. proteamaculans* isolate. All recombinants were validated by PCR using inward-facing primers positioned external to the recombined region, with the primers 143F and 143R used for the *tcAB* recombinants, 1516F and 1516R for *afpX15* to *afpX16a* AfpX recombinants, and BGLIIF and BGLIIR for S. entomophila Afp recombinants (Table S6), with the resultant amplicons then sequenced.

### Host-range bioassay assessments.

Bioassays were carried out using field-collected third-instar larvae of the New Zealand grass grub (*C. giveni*), manuka beetle (*Pyronota* spp.), and chafer beetle (*Odontria* sp.). Larvae of the manuka beetle species *P. setosa* were differentiated from those of *P. festiva* and *P. laeta* by differences in the rostral seta pattern of this species. Based on the known prevalence of *P. festiva* over that of *P. laeta*, and because these two species can be visually differentiated only at the adult stage, all *Pyronota* larvae other than *P. setosa* were assumed to be *P. festiva*.

For bioassays, *C. giveni* and *Odontria* sp. larvae were individually fed carrot cubes measuring ∼3 mm^3^ treated with a single bacterial isolate. Carrot cubes used to deliver bacteria to the smaller *P. festiva* and *P. setosa* larvae measured ∼1.5 mm^3^. To prepare the bacterial inoculum, carrot cubes were rolled in a lawn of bacteria cultured overnight on solid LB agar medium, resulting in approximately 4 × 10^8^ bacterial cells per carrot cube. Twelve larvae were used for each treatment, and the assay was carried out in a randomized block design using two blocks of six larvae. Larvae were fed treated carrot on day 1 and then transferred to fresh trays containing untreated carrot on days 3 and 6. The onset of disease symptoms, including changes in coloration from a healthy gray to amber or other atypical coloration as well as cessation of feeding, was monitored on days 3, 6, and 12 postchallenge. Tested isolates were considered virulent if at least five of the treated larvae showed disease symptoms by day 12 and if at least nine of the larvae from the negative control remained healthy. Larvae were then scored as the percent diseased or dead. S. entomophila strain 626 and *S. proteamaculans* strain AGR96X were used as positive controls in all bioassays, and untreated carrot was used as a negative control. Bioassays were undertaken in triplicate at independent times. Due to their accessibility and vigor, *C. giveni* larvae were used as the standard test larvae for mutation-based bioassays.

### Statistical analysis.

Bioassay data were assessed at 12 days postchallenge using a generalized linear model with group-specific binomial distributions through a logit link function. For assessment of the relative virulence of IDIA compared with A1MO2, each variable was compared at days 3, 6, and 12 posttreatment using Fisher’s binomial exact test (62).

Cumulative diseased and mortality rates over the bioassay duration were compared between the IDIA and A1MO2 strains using the log-rank test. Because the untreated control mortality was not zero, the cumulative diseased and mortality rates were adjusted for control mortality prior to their comparison using Abbott’s formula (63). Variability of bioassays of MH5 and 28F was calculated at 12 days posttreatment using measures of spread (62). All analyses were performed using Minitab version 16.
