# Supplementary material for: Identification of Diverse Toxin Complex Clusters and an eCIS Variant in Serratia proteamaculans Pathovars of the New Zealand Grass Grub (Costelytra Giveni) and Manuka Beetle (Pyronota Spp.) Larvae
Source: Microbiol Spectr. 2021 Oct 20;9(2):e01123-21. doi: 10.1128/Spectrum.01123-21 (PMC8528098; doi:10.1128/Spectrum.01123-21)
Supplement: SUPPLEMENTAL FILE 1 — Supplemental material. Download SPECTRUM01123-21_Supp_1_seq4.pdf, PDF file, 1.1 MB [file spectrum01123-21_supp_1_seq4.pdf]

Supplemental files

Identification of diverse toxin complex clusters and an eCIS variant in *Serratia proteamaculans* pathovars of the New Zealand grass grub (*Costelytra giveni*) and manuka beetle (*Pyronota* spp.) larvae.

Mark RH Hurst[1,2#], Amy Beattie[1], Aurelie Laugraud[3], Richard Townsend[1], Lesley Sitter[1,2], Chikako van Koten[3] and Lincoln Harper[4]

[1]Resilient Agriculture, AgResearch, Lincoln Research Centre, Private Bag 4749, Christchurch 8140, New Zealand

[2]Bio-Protection Research Centre, Lincoln, Christchurch, New Zealand

[3]Knowledge & Analytics, AgResearch, Lincoln Research Centre, Private Bag 4749, Christchurch 8140, New Zealand

[4]Curtin University, Centre for Crop and Disease Management, School of Molecular and Life, Bentley, WA, Australia

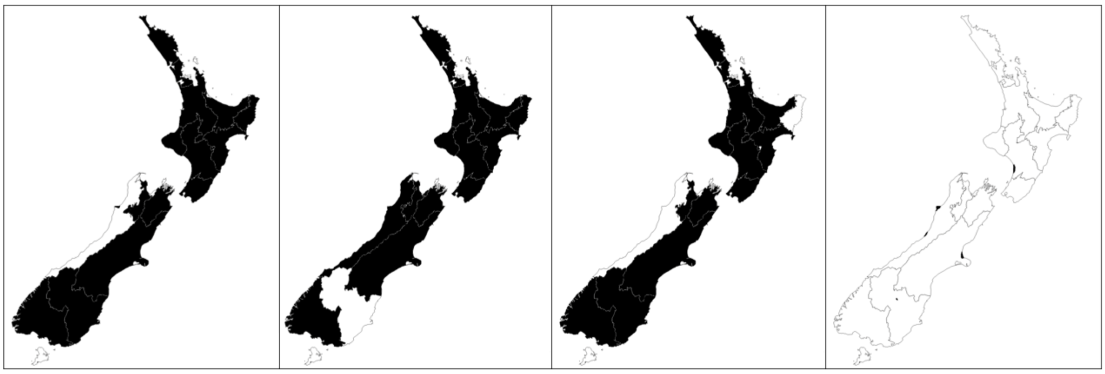

Supplemental Fig. S1. Distribution (black shading) of *C. giveni* and the *Pyronota* species (*P. festiva* white area -no collection data available), *P. laeta,* and *P. setosa*, throughout New Zealand, based on (1, 2).

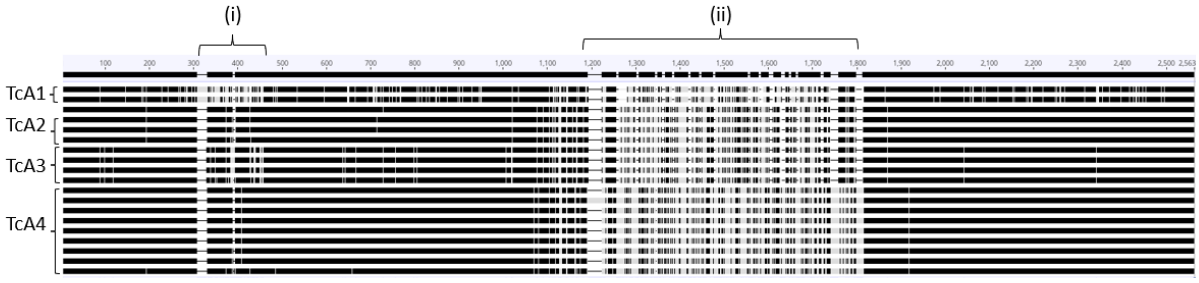

Supplemental Fig. S2 schematic of amino acid alignment of TcA1-TcA4 variants. (i) and (ii), denote the two regions of amino acid divergence across the TcA which relative to SppA spanned amino acid residues 306-429 and 1027-1658 respectively. Black shade denotes shared amino acid identity. Refer to Fig. 4 for schematic depiction of the TcA1-TcA4 components in relation to their associated Tc clusters.

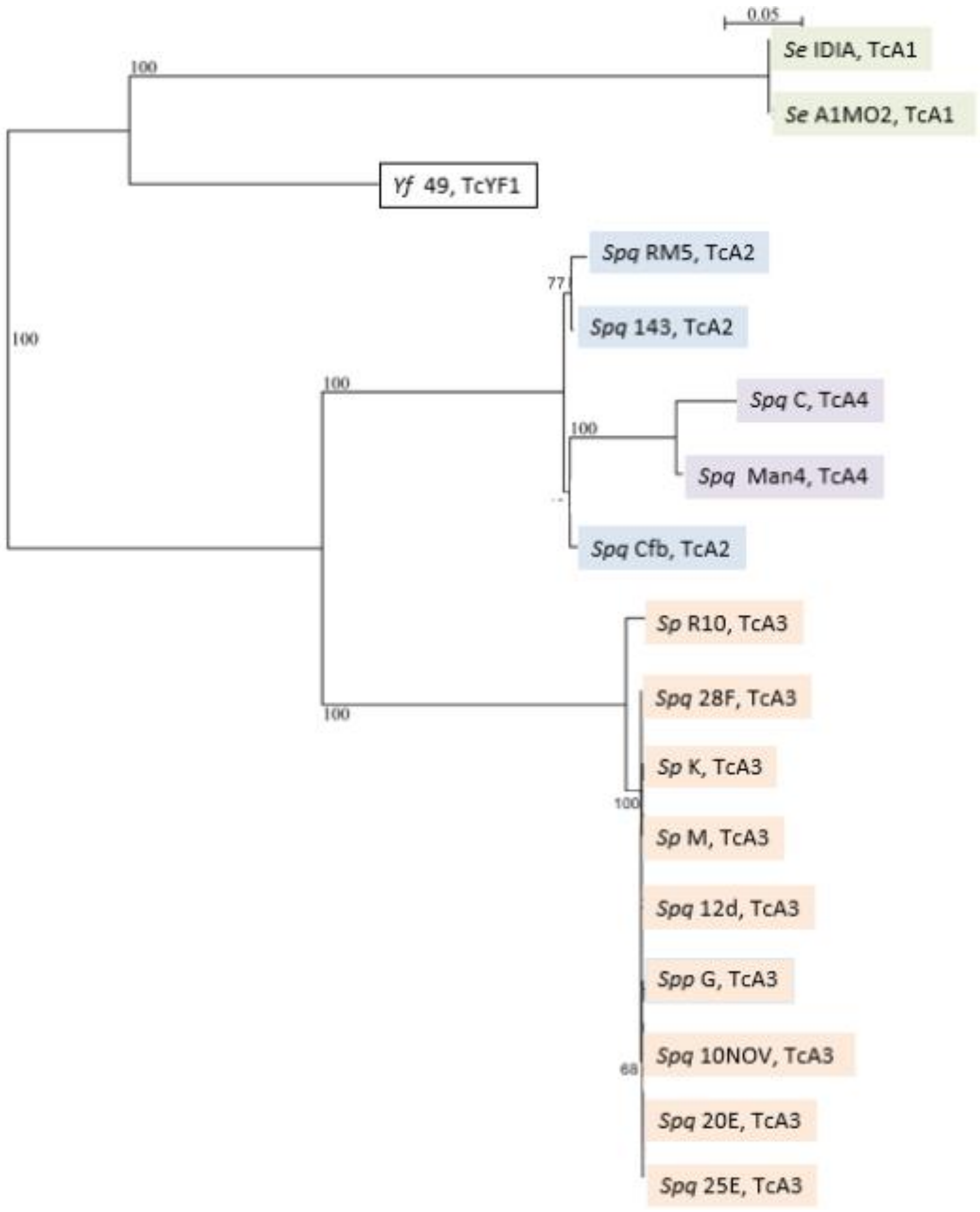

Supplemental Fig. S3. Maximum-likelihood phylogenetic tree based on the *S. entomophila* and *S. proteamaculans* TcA amino acid sequence, using the LTR model, performed at 1000 bootstraps. Numbers denotes bootstrap values greater than 50%. *Sp*, *Serratia proteamaculans*, *Spq*, *S. proteamaculans* subsp. *quinovora* and *Spp S. proteamaculans* subsp. *proteamaculans*. Color coded TcA1-4 components are indicated. The *Yersinia frederiksenii* isolate 49, TcA orthologue TcYF1 is indicated.

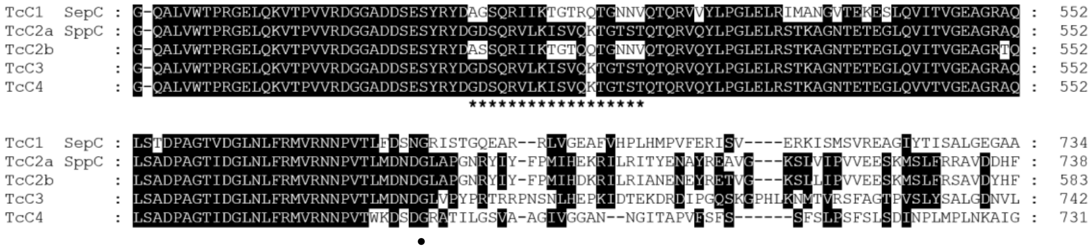

Supplemental Fig. S4. Amino acid alignments of *Serratia* derived TcC1-TcC4 variants. (•) denotes position of the conserved glycine residue which demarcates the Rhs conserved *N*-terminus from the variable carboxyl terminus. Asterisk denotes 18-amino acid region difference between Tcc2a and Tcc2b shared with TcC1.

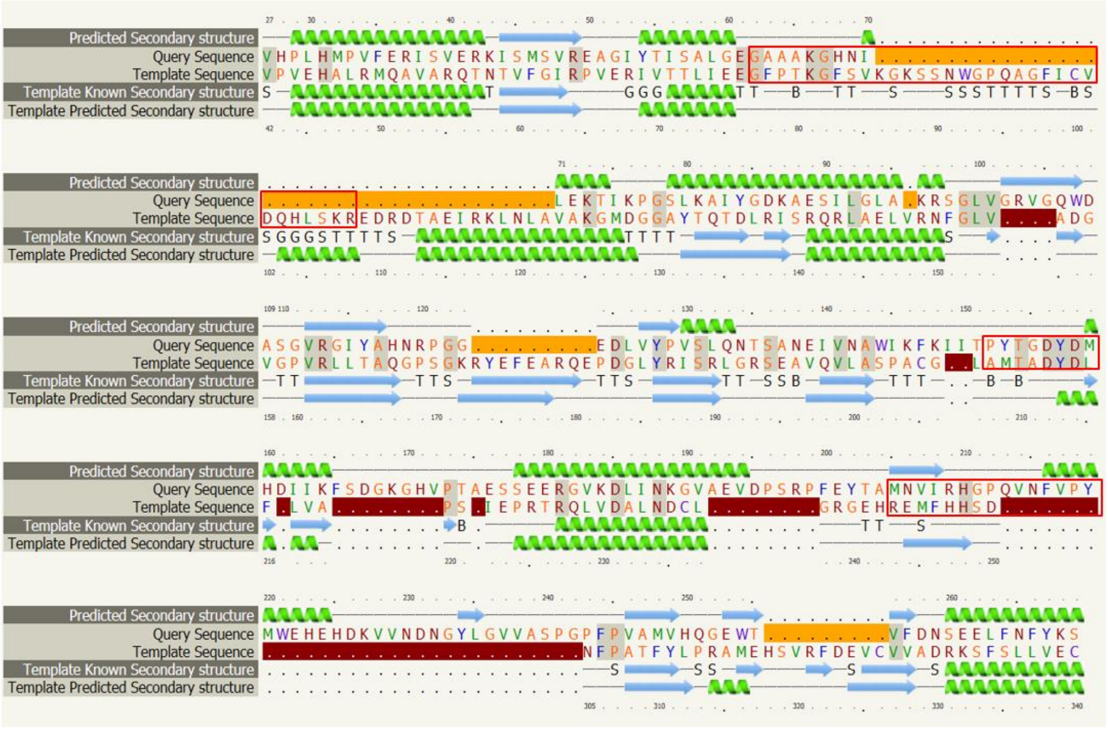

Supplemental Fig. S5 Secondary structure and disorder prediction derived from Phyre[2] (2). Red boxes denote amino acid motifs of the *Pseudomonas aeruginoas* type III secretion nucleotidyl cyclase toxin ExoY (3)

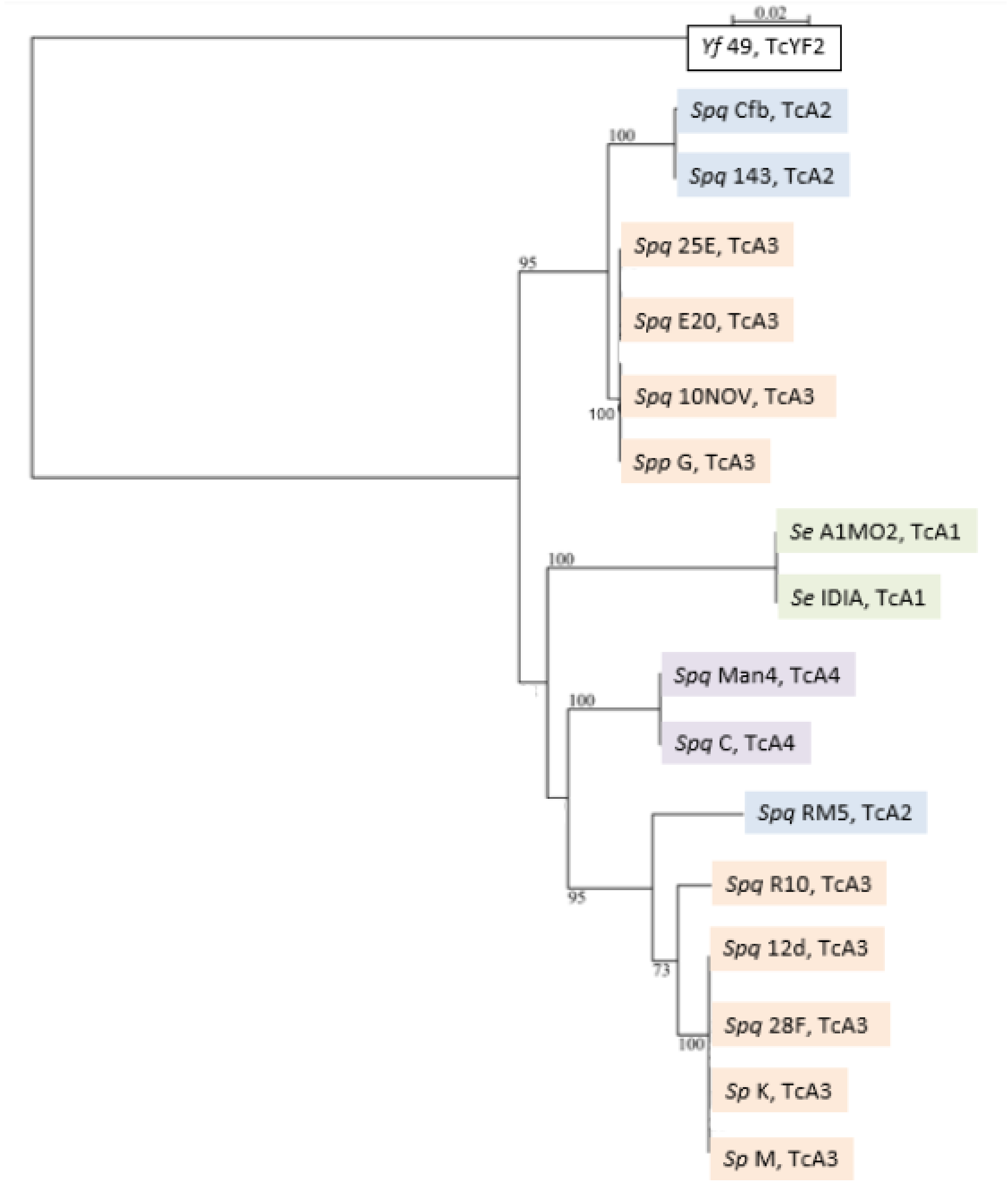

Supplemental Fig. S6. Maximum-likelihood phylogenetic tree based on the *S. entomophila* and *S. proteamaculans* TcB amino acid sequences using the LTR model, performed at 1000 bootstraps. Numbers denotes bootstrap values greater than 50%. *Sp*, *Serratia proteamaculans*, *Spq*, *S. proteamaculans* subsp. *quinovora* and *Spp S. proteamaculans* subsp. *proteamaculans*. Color coded TcA1-4 components are indicated. The *Yersinia frederiksenii* isolate 49 TcB orthologue TcYF2 is indicated.

```
Se :MSPSPLTGAALMETKMKIHYQVAAVVLTGVMVWGLSHWRYTVGYHAADT----QWQQRQAEQERADALAL :66
As :----------MVAIPQSRVLPFLAGALVIAALAGAGVALYQSGHSDGEEGERKTWQAKWNEESARLATAR :60
Yp :-------------MMWRYWKIVLVAGLVVAAIAAFSHWRWQAGFNEAEG----KWQQRWSQRDADDATAL :53
Et :----------------MKPTLTVAVVLCGLAVWWFSQQRYMAGYSRADA----EWSLKWKQRDADDATAL :50
Sr :-------------MGLSRWKVIVCLVLAAAAVWGFSHWRYSAGYGDADQ----HWREEWAQRDARDAAAQ :53
Ec :-------------MGLSRWKVIVCLVLAAAAVWGFSHWRYSAGYGDADQ----HWREKWAQRDARDATAL :53
Er :-------------MLSNIWKPLAIISLVALSLWGLSTWRYAAGYAAGKRLAEQVWQLKWETRNRDEESAR :57
Sp :-----------MIKLASIIFKVGGVVLLGATVGVFSHWRYTAGKESANT----VWQSRWDKRNIADVELM :55

Se :LAAETRERKWEQQRQTDMNKVAIHAEEELAAARDAAADAQRTGQRLQHTVTTLQRQLASRETRRLSAATA :136
As :TKAEQEAREEENRRQAEIDEVRDHAQEQIAQAQADAAAAGVESGRLREQA----RRLAARASQCASNPST :126
Yp :AKRQTEARNEEQRRQNEINEIRNSAAQQLAVVLADASTARAAADRLHDSAEILARRLADRERTC--HPAT :121
Et :AERQAEARKEEQRRQGEIDEIRKQTSQQLAAVQADVDRARTASRGLHDRAGKLARQLAERERTC--GAGT :118
Sr :AQRQAEARAEEQRRQGEIDAIRKQASQQLAGVQADADRARAASRGLHDRADKLARQLADRERAC--GAGT :121
Ec :AQRQAEARTEEQRRQGEIDAIRKQTSQQLAGVQAAADRASAVSRGLHAKADELARRLADRERAC--GAGT :121
Er :ANRERGERAEEQRRWQAMIKVKQNADQQLEQIKADAARSTADAERLRRTLSQLRQQLADRSPCR--VSTA :125
Sp :ARYQREAREEEQRRIKAIIQVNEDANLQLNAARADAARAKSVIISLRNTVRYMQQKLAADNAAG--VSAT :123

Se :IGTDDLGGQPGVLFAELFRRADQRAGELAAYADRTRVKWQACGRAYQAATHEAEK---------- :191
As :AQGSPATGQPAMVLADLLSRADERAGELAAAYDRARASGLACERAYNSLRTSTMKPRP------- :184
Yp :PGAGQTAGSGAVLLAELFRRADERAGELAAAADEARTRGLACENAYNALKNW------------- :173
Et :PGRSEAETSGAILLADLFRRADKRAGELAGEADEARARGLACEAAYDSISRLR------------ :171
Sr :PGRSEAETSGAVLLADLFRRADDRAGQLAKDADEARARGLACEAAYDAVKSGRDK---------- :176
Ec :PGRSEAKASGAALLADLFKRADERAGELAREADEARARGLACEAAYDAVKSGGNK---------- :176
Er :GGASPASAAAGFLFADVLGESLQRNAALAAYADRTRAAGLACERLYDAVTQSRAP---------- :180
Sp :VGKRQAGGDSHLLLADMFAESLKRNQQLAAYADRARIRGRACERAYDAITQSAAGAVKTSYNIER :188
```

Supplemental Fig. S7 Amino acid alignment of selected Gp55 orthologues from *S. entomophila* (Se, WP_010895740); *Aeromonas salmonicid* (As, WP_073531742, *Yersinia pestis* strain I-2638 (Yp, AKT73144); *Erwinia tracheiphila* (Et, AXF77457); *Salmonella enterica* subsp. enterica strain RM11060 (Sr, ATI92601); *Escherichia coli* strain AR_0128 (Ec, ARW90342); *Edwardsiella tarda* strain KC-Pc-HB1 (Er, ATI65634) and *Sodalis praecaptivus*.

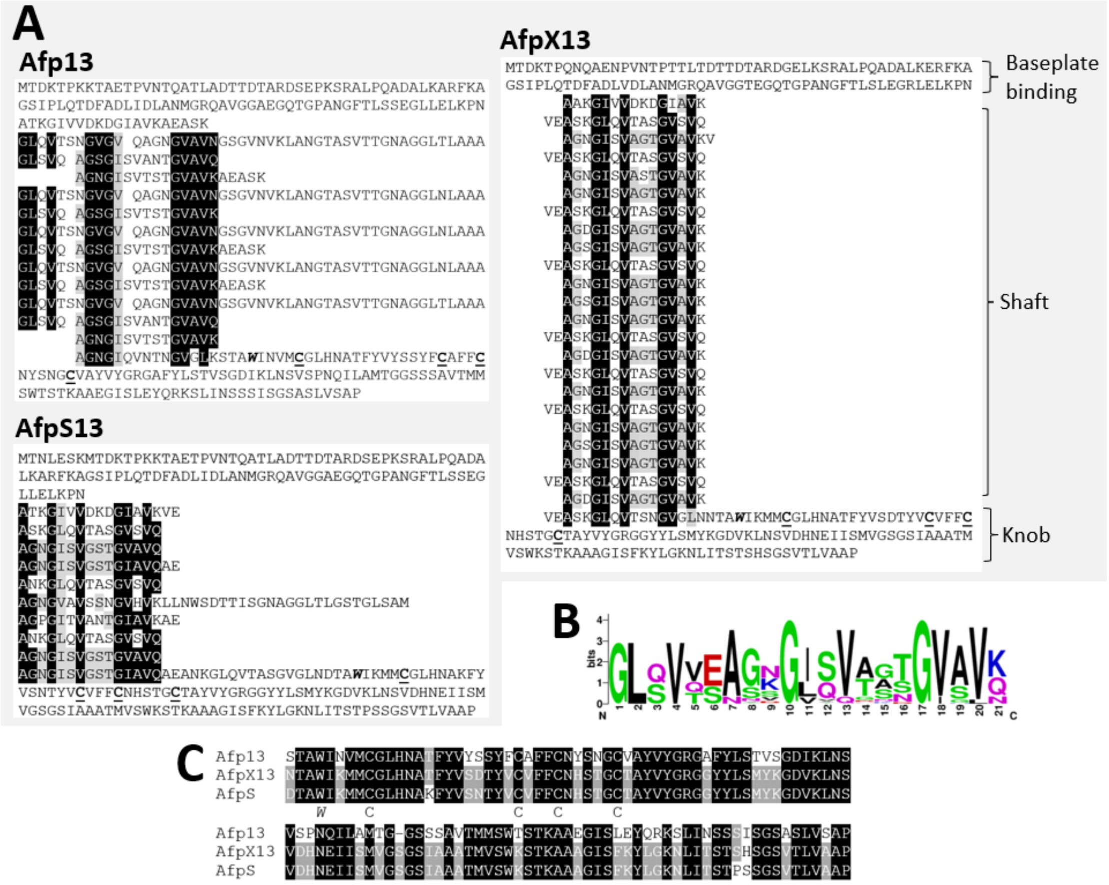

Supplemental Fig. S8 A) Amino acid motifs in the Afp tail fibers of Afp13, AfpX12 and AfpS13. The 418 amino acid residue AfpS13 (11 shaft repeats); 590 amino acid residue AfpX13 (24 shaft repeats) and the 636 amino acid residue Afp13 (14 shaft repeats). After each fiber repeat region there is a conserved tryptophan residue (***W***) followed by a series of four cysteine residues (<u>**C**</u>) located in the predicted tail fiber knob region. **B)** WebLogo 3 alignment showing conserved shaft amino acid repeat shared between Afp13, AfpX13 and AfpS13. **C)** Amino acid alignment of the predicted Afp, AfpX AfpS tail fiber knob region.

Supplemental Table S1 Variability of percent diseased and dead, 12 days after maximum challenge of *C. giveni* larvae with 4x10^8 cells of either 28F or MH5 over three independent bioassays

Statistic	28F	MH5
Mean ± SEM	75.0 ± 8.7	76.4 ± 8.5
Standard deviation	19.5	20.7
Minimum - Maximum (range)	58.3-100 (41.7%)	50-100 (50%)

Supplemental Table S2 Synteny orthologues of flanking Gp55 ORFs to translated amino acid sequences in the database detected using BlastP

	Accession number	Predicted function
Edwardsiella tarda strain KC-Pc-HB1		
	ATI65633.1 (GI:1253310454)	antA/AntB anti-repressor
Gp55 orthologue †	ATI65634.1 (GI:1253310455) (180) 37/51//175 (2 -172)‡	hypothetical protein
	ATI65635.1 (GI:1253310456)	hypothetical protein
Salmonella enterica subsp. enterica strain RM11060		
	ATI92600.1 (GI:1253337759)	muraminidase
Gp55 orthologue †	ATI92601.1 (GI:1253337760) (176) 38/52//163 (7-169)‡	hypothetical protein
	ATI92602.1; GI:1253337761	phage tail protein
Yersinia pestis strain I-2638 plasmid pTP33		
	AKT73143.1 (GI:908777934)	phage-related lysozyme
Gp55 orthologue †	AKT73144.1 (GI:908777935) (173) 40/55//163 (7-169)‡	hypothetical protein
	AKT73145.1 (GI:908777936)	Integrase
Erwinia tracheiphila strain MDcuke		
	AXF77458.1 (GI:1433534066)	lysozyme
Gp55 orthologue †	AXF77457.1 (171) 37/51//172 (1-172)‡	hypothetical protein
	AXF77456.1 (GI:1433534064)	phage tail protein
Escherichia coli strain AR_0128		
	ARW90343.1 (GI:1202200120)	TIGR02594 family protein
Gp55 orthologue †	ARW90342.1 (176) 36/51//170 (7-176)‡	hypothetical protein
	ARW90341.1 (GI:1202200118)	phage tail protein

† *Serratia entomophila* (WP_169558214.1) 176 amino acid residue Gp55 orthologue.

‡ % identity/% similarity (amino acid residues of target sequence). Refer to Supplemental Fig. S7 for amino acid alignments.

Supplemental Table S3 Source locations of *S. entomophila* and *S. proteamaculans* isolates reported by Dodd *et al*., (4) [a]

Species isolate	New Zealand source location	Farm type
S. entomophila		
167	Methven, Canterbury	Hill country
168	Lauriston, Canterbury	Low land
203, 207, 213	Temuka, Canterbury	Low land
210	Oamaru, North Otago	Low land
176, 291, 292, 345, 398	Ashbury, Canterbury	Low land
307	Takapau, Hawkes Bay	Low land
369	Riversdale, Southland	Low land
402	Ashbury, Canterbury	Low land
465, 1100, 1347	Hawkes Bay	Low land
480	Tihoi, Taupo	Hill country
673	Eltham, New Plymouth	Low land
S. proteamaculans		
142, 387	Tihoi, Taupo	Hill country
145	Waimate, South Canterbury	Hill country
299, 310	Motueka, Tasmin	Low land
341	Ashbury, Canterbury	Low land
376	Taupo	Hill country
1048	Pendarves, Canterbury	Low land
1071	Canterbury	Low land
1129	Hawkes Bay	Low land

[a] all isolates defined as causing a chronic disease by Dodd *et al.,* (4)

Supplemental Table S4 Cumulative percent diseased and adjusted mortality over 12 days after maximum challenge of *C. giveni* larvae with the *S. entomophila* isolates IDIA and A1MO2.

	IDIA	A1MO2
Day	Disease + mortality (%) ± SE (%)	Disease + mortality (%) ± SE (%)
Day 3	93.8 ± 2.5	78.1 ± 4.2
Day 6	99.0 ± 1.0	94.8 ± 2.3
Day 12	100	100

Supplemental Table S5 Strains and plasmids used in this study

**Strain or plasmid**	**Relevant characteristics**	**Reference or source**
Escherichia coli		
DH10B	F- *mcr*A Δ*mrr-hsd*RMS-*mcr*BCΔ80d *lacZ*ΔM15 Δ*lacX74 endA1 recA1 deo*RΔ*ara, leu* 7697 *ara*D139 *gal*U *gal*K *nup*G *rps*L Δ$^-$.	(5)
ST18	*hemA* λ *pir* lysogen of S17-1: *pro*, *res*$^-$, *mob*$^+$, *recA*$^-$ derivative of *E. coli* 294 with Rp4-2 (TcΔmu) (KmΔTn7) in the chromosome	(6)
**TcAB mutant strains**[a]		
20EΔTCAB	*tcA*$^-$, *tcB*$^-$, Sp^R	This study
CfbΔTCAB	*tcA*$^-$, *tcB*$^-$, Sp^R	This study
RM5ΔTCAB	*tcA*$^-$, *tcB*$^-$, Sp^R	This study
Man4ΔTCAB	*tcA*$^-$, *tcB*$^-$, Sp^R	This study
CΔTCAB	*tcA*$^-$, *tcB*$^-$, Sp^R	This study
12DΔTCAB	*tcA*$^-$, *tcB*$^-$, Sp^R	This study
MΔTCAB	*tcA*$^-$, *tcB*$^-$, Sp^R	This study
KΔTCAB	*tcA*$^-$, *tcB*$^-$, Sp^R	This study
10NOVΔTCAB	*tcA*$^-$, *tcB*$^-$, Sp^R	This study
25EΔTCAB	*tcA*$^-$, *tcB*$^-$, Sp^R	This study
28FΔTCAB	*tcA*$^-$, *tcB*$^-$, Sp^R	This study
GΔTCAB	*tcA*$^-$, *tcB*$^-$, Sp^R	This study
R10ΔTCAB	*tcA*$^-$, *tcB*$^-$, Sp^R	This study
IDIAΔAFP	*afp*$^-$ Kn^R	This study
IDIAΔTCAB	*tcA*$^-$ *tcB*$^-$ Sp^R	This study
IDIAΔTCABΔAFP	*afp*$^-$, *tcA*$^-$, *tcB*$^-$, Sp^R, Kn^R	This study
**Afp mutant strains**		
AGR96XΔ1516	*afpX*$^-$ Kn^R	(7)
2009Δ1516	*afp*X15$^-$, *afp*X16$^-$, Kn^R	This study
LCΔ1516	*afp*X15$^-$, *afp*X16$^-$, Kn^R	This study
1AΔ1516	*afp*X15$^-$, *afp*X16$^-$, Kn^R	This study
MH5Δ1516	*afp*X15$^-$, *afp*X16$^-$, Kn^R	This study
SpfΔ1516	*afp*S15$^-$, *afp*S16$^-$, Kn^R	This study
**Plasmids**		
pADAP	Amber disease associated plasmid, *sep*$^+$ *afp*$^+$	(8)
pADAPX	pADAP variant, *afpX*$^+$	(7)
pGEM-Teasy	PCR cloning vector, Ap^R	Promega corporation
pGEM143A	4062-bp amplicon TCAB containing the 3' region of *tcA* (*spp*A) and the 5' region of *tcB* (*spp*B) cloned into pGEM Teasy, Ap^R	This study
pGEM143SPRV	pGEM143A containing the Spectinomycin resistant amplicon SPRV inserted in the MscI and SmaI sites deleting 293-bp 3' *tc*A and 22 bp 5' *tc*B, Ap^R Sp^R	This study

pJP5608	Suicide plasmid, Tc^R	(9)
pJP143TCAΔBSP	BamHI fragment derived from pGEM143SPRV cloned into the BamHI site of pJP5608, Tc^R Sp^R	This study
pJP5608ΔAFP1516a	pJP5608 based vector used to delete 1745-bp region corresponding to amino acid residue 205 of Afp15 to amino acid residue 70 of Afp16, Tc^R Kn^R	(7)
pMH52ΔBglII	pLAFR3 based plasmid used to delete 16,064-bp fragment encompassing the *afp* genes *afp*2-*afp*15, Tc^R Kn^R	(10)

[a] Δ denotes internal deletion

Supplemental Table S6 Primers used in the study

**Primer name**	**Template target**	**Primer sequence 5′–3′**	**Amplicon size (bp)**
BOXA1R		CTACGGCAAGGCGACGCTGACG	(49)
pADAP and virulence specific primers			
RepAF<br>RepAR	pADAP<br>*repA*	TGCAGGGGAACGATCTTCTTGAGG<br>GCCCCACTTTCTTGTACCATCCAG	893
Afp8F<br>Afp8R	*afp8*	GGACATAGCGGGACAGCGCAGCAC<br>ACTCAGGGTGATACTCTCGCCCG	909
Afp18F<br>Afp18R	*afp18*	GAACGTGCAGAATCTCAGCCTGAG<br>AAGCGTAGCGTTCATCGAAGCCAG	771
AfpX17F<br>AfpX17R	*afpX17*	CGACTAACACACCACAGTTACAGC<br>ACGCATGATCATTTGCGCTTCCGG	2600
AfpX18F<br>AfpX18R	*afpX18*	CAACTGGAGCAGTTCGAGCTGGC<br>GTCATAAGACTCATGCCAAGAGTC	805
TcA1F<br>TcA1R	TcA1, *sepA*	CCTCCTCACTGTTATCTTATGATGC<br>ATGCCCGTGGCCTTGTATAGGCGG	554
TcA2F<br>TcA2R	TcA2, *sppA*	TGGGAGTATAAATCCGCTGTATGGC<br>GCAAGGTCACCAATAATGATCCGG	812
TcA3F<br>TcA3R	TcA3	CGTATTGGTCAGACCTCAATGATG<br>CAACACAGTAGTGTTCAGTCCGC	2120
TcA4F<br>TcA4R	TcA4	ATCCCCAGTTAGCCGGTGCCGCG<br>GATACAGGATTTCATCGGAAGCGT	1052
TcC1F<br>TcC1R	TcC1, *sep*C	CAAGAAGTTCAGCATGCCGAGGAG<br>TCAATGAGCGTAAGGGAAGCTGGC	600
TcC2aF<br>TcC2aR	TcC2a,<br>*spp*C	CAGGCGTGCAGTTGATGATCACTT<br>GACTATGGGGTCACCCGCCTTATCT	645
TcC2bF<br>TcC2bR	TcC2b	GGAGTGCGGTTGATTATCATTTTTCAC<br>CCTTATTTCCAAAAACACGCTTGA	614
TcC3F<br>TcC3R	TcC3	TTGCAGGAACCCCTGTTAGTCTC<br>ACCAGGTCGATCACTACCCGCCTCTC	587
TcC4F<br>TcC4R	TcC4	TCGGTAATGCGGCCAGTGGTGG<br>CTCCTGCCATAGTGCTTACAGCC	450
**Targeted mutagenesis**			
**Amplicon name**	**Primer name**	**Primer sequence 5′–3′ †**	**Amplicon size (bp)**
SPRV<br>Spectinomycin cassette	SPRVF<br>SPRVR	AAAGATATCGCCCGTTCCATACAGAAGCTGGG<br>AAAGATATCCGACATTATTTGCCGACTACCTTG	1379
KNB<br>Kanamycin cassette	KNBF<br>KNBR	AAAGGATCCTAAGCCAGTATACACTCCGCTAGC<br>AAAGGATCCAGTGTTACAACCAATTAACCAATT	1201
TCAB	ABTCF<br>ABTCR	AAAGGATCCTGCAGACTTTACACCTGTCGG<br>TTTGGATCCAGGTCACAGCATCCGGATCATCC	4062
Validation primers			
TcAB deletion	143F<br>143R	GTTGGCAAGCGACGAACGGG<br>GCTTCCACCTGGGCTTATCCCCAG	2729
AfpX15Δ16 validation	1516F<br>1516R	GAGTCGTTGTTTACAACGCCAGG<br>GTGCGCATCTGCATTTACCGG	4814
Afp BglII	BGLIIF<br>BGLIIR	TTTACCCGTGGGGCACCCTGGC<br>GTGAAACCTTAATGTGCTTCGGCC	832
Afp BglII	BGLIIF<br>BGLIIR	CCGCTTGCTGACCAAACGTACGG<br>CGGTTATCCCGGTGCCAGCCAC	

†Underscore denotes restriction enzyme site

References

1. Thomson NA, Miln AJ, Kain WM. 1979. Biology of manuka beetle in Taranaki. Proc NZ Weed Pest Contr Conf 32:80-85.
2. Brown JG, 1963. Biology and taxonomy of the genus *Pyronota* Bois. (Melolonthinae, Coleoptera). University of Canterbury, New Zealand Lincoln College.
3. Khanppnavar B, Datta S. 2018. Crystal structure and substrate specificity of ExoY, a unique T3SS mediated secreted nucleotidyl cyclase toxin from *Pseudomonas aeruginosa*. Biochim Biophys Acta 1862:2090-2103.
4. Dodd SJ, Hurst MRH, Glare TR, O'Callaghan M, Ronson CW. 2006. Occurrence of Sep insecticidal toxin complex genes in *Serratia* species and *Yersinia frederiksenii*. Appli Environ Microbiol 72:6584-6592.
5. Lorow D, Jesse J. 1990. Max efficiency DH10B™ a new host for cloning methylated DNA. Focus 12:19-20.
6. Thoma S, Schobert M. 2009. An improved *Escherichia coli* donor strain for diparental mating. FEMS Microbiol Lett 294:127-132.
7. Hurst MRH, Beattie A, Jones SA, Laugraud A, van Koten C, Harper L. 2018. *Serratia proteamaculans* strain AGR96X encodes an antifeeding prophage (Tailocin) with activity against grass grub (*Costelytra giveni*) and manuka beetle (*Pyronota* species) larvae. Appl Environ Microbiol 84:e02739-17.
8. Glare TR, Corbett GE, Sadler AJ. 1993. Association of a large plasmid with amber disease of the New Zealand grass grub, *Costelytra zealandica*, caused by *Serratia entomophila* and *Serratia proteamaculans*. J Invertebr Pathol. 62:165-170.
9. Penfold RJ, Pemberton JM. 1992. An improved suicide vector for construction of chromosomal insertion mutations in bacteria. Gene 118:145-146.
10. Hurst MR, Glare TR, Jackson TA. 2004. Cloning *Serratia entomophila* antifeeding genes a putative defective prophage active against the grass grub *Costelytra zealandica*. J Bacteriol 186:5116-5128.
